# A mechanism for temporary bioadhesion

**DOI:** 10.1073/pnas.1814230116

**Published:** 2019-02-19

**Authors:** Julia Wunderer, Birgit Lengerer, Robert Pjeta, Philip Bertemes, Leopold Kremser, Herbert Lindner, Thomas Ederth, Michael W. Hess, David Stock, Willi Salvenmoser, Peter Ladurner

**Affiliations:** ^a^Institute of Zoology, University of Innsbruck, 6020 Innsbruck, Austria;; ^b^Center of Molecular Bioscience Innsbruck, University of Innsbruck, 6020 Innsbruck, Austria;; ^c^Biology of Marine Organisms and Biomimetics, Research Institute for Biosciences, University of Mons, 7000 Mons, Belgium;; ^d^Division of Clinical Biochemistry, Biocenter, Innsbruck Medical University, 6020 Innsbruck, Austria;; ^e^Division of Molecular Physics, Department of Physics, Chemistry and Biology, Linköping University, 58183 Linköping, Sweden;; ^f^Division of Histology and Embryology, Innsbruck Medical University, 6020 Innsbruck, Austria;; ^g^Institute for Material Technology, University of Innsbruck, 6020 Innsbruck, Austria

**Keywords:** flatworms, bioadhesion, Platyhelminthes, bioadhesive, detachment

## Abstract

The flatworm *Macrostomum lignano* features a duo-gland adhesive system that allows it to repeatedly attach to and release from substrates in seawater within a minute. However, little is known about the molecules involved in this temporary adhesion. In this study, we show that the attachment of *M. lignano* relies on the secretion of two large adhesive proteins, *M. lignano* adhesion protein 1 (Mlig-ap1) and Mlig-ap2. We revealed that both proteins are expressed in the adhesive gland cells and that their distribution within the adhesive footprints was spatially restricted. RNA interference knockdown experiments demonstrated the essential function of these two proteins in flatworm adhesion. Negatively charged modified sugars in the surrounding water inhibited flatworm attachment, while positively charged molecules impeded detachment. In addition, we found that *M. lignano* could not adhere to strongly hydrated surfaces. We propose an attachment–release model where Mlig-ap2 attaches to the substrate and Mlig-ap1 exhibits a cohesive function. A small negatively charged molecule is secreted that interferes with Mlig-ap1, inducing detachment. These findings are of relevance for fundamental adhesion science and efforts to mitigate biofouling. Further, this model of flatworm temporary adhesion may serve as the starting point for the development of synthetic reversible adhesion systems for medicinal and industrial applications.

## Significance

Synthetic adhesives are widely used in our daily lives, in medicine and industry. These man-made glues contain toxic or carcinogenic components. In contrast, biological adhesives produced by animals and plants are nontoxic and tissue-compatible, and are able to function under wet conditions. However, little is known about the mechanisms underlying biological adhesives. We characterized adhesion and release in our model system *Macrostomum lignano*. We used a state-of-the-art toolbox to identify the involved adhesive and release molecules. We aim for understanding the fundamental mechanisms that mediate adhesion and release in flatworms, with the future goal of generating a flatworm-derived biomimetic glue that can be applied in biomedicine and industry.

Bioadhesion is the attachment of an organism to a surface using natural macromolecules. An increasing number of studies have focused on the investigation of marine biological adhesives and the development of biomimetic counterparts (1–3). Bioadhesives could be a nontoxic, biodegradable, and yet strong-adhering alternative to the medical adhesives currently in use (4). Biological attachment is a common feature among several marine invertebrate species (5). It is essential for feeding, locomotion, defense, mating, and to prevent dislodgement (6). Bioadhesion can be divided into permanent and temporary attachment systems (7). To date, most scientific advances have been made in the characterization of permanent adhesives, such as those of mussels, tubeworms, and barnacles (8–10). In contrast to permanent adhesion, animals with temporary adhesive systems can voluntarily detach from a substrate. After detachment, the secreted adhesive material stays permanently attached to the surface as so-called footprints. Such systems are found in echinoderms (7, 11) and flatworms (12–14). To date, reversible adhesion and its related secretions are poorly understood, and only certain components have been identified (15–18).

Free-living marine and freshwater Platyhelminthes use a duo-gland adhesive system to adhere and release (13, 19). Their adhesive system consists of dozens to hundreds of adhesive organs. Each adhesive organ comprises three cell types: the adhesive gland, a releasing gland, and a modified epidermal cell, called an anchor cell (13, 14). However, little is known about the composition of the adhesive substances. Our model system, *Macrostomum lignano*, lives between the sand granules of the intertidal zone. In its natural environment, *M. lignano* can attach and release several times to any substrate within a single minute (12, 20). A broad molecular toolbox for *M. lignano* has been established, including whole-mount in situ hybridization, RNA interference (RNAi), and transgenesis (20–33), allowing adhesion studies not feasible in other adhering species.

In this study, we present a characterization of the adhesive substances used for temporary adhesion in a flatworm species. We identified two large adhesion proteins and analyzed their secretion upon attachment. Both proteins showed particular characteristics, such as high cysteine content, large repetitive regions, and a number of known protein–carbohydrate and protein–protein interaction domains. The essential function of the proteins in the adhesion process was corroborated with RNAi-mediated knockdown. We performed attachment assays and tested different molecules and surfaces regarding their interference with attachment and release. In addition, we showed that negatively charged sugars were able to inhibit the adhesion, while positively charged molecules interfered with the natural detachment of the flatworm. These results were incorporated into a model for the attachment and release of *M. lignano*. Our findings provide a better understanding of an effective temporary adhesion system with great biomimetic potential.

## Results

### Characterization and Localization of Adhesive Transcripts and Proteins.

**Fig. 1.**
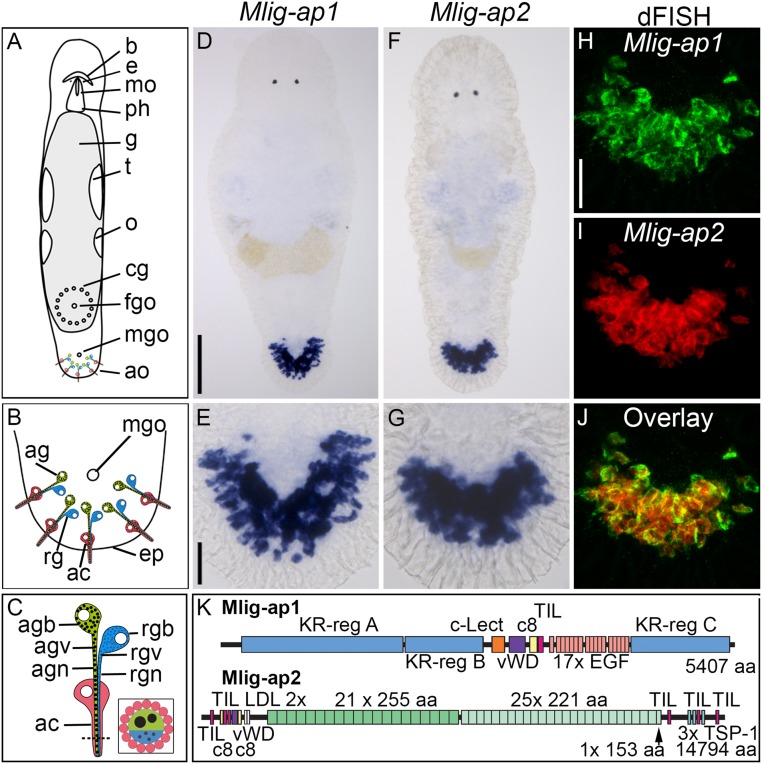
Location, expression, and organization of *M. lignano* adhesive proteins. (*A*–*C*) Schematic drawing of adult *M. lignano* with detailed structure of adhesive organs. (*D*–*J*) Expression of *Mlig-ap1* and *Mlig-ap2* mRNA visualized with colorimetric WISH (*D*–*G*) and dFISH (*H*–*J*). Note the coexpression in the same cells of both mRNAs in the overlay. (*K*) Schematic drawing of the protein structure of the two adhesive proteins. Protein domains: aa, amino acids; c8, domain of eight conserved cysteines; c-Lect, c-type lectin domain; EGF, epidermal growth factor-like domain; LDL, low-density lipoprotein receptor-like domain; TIL, trypsin inhibitor-like domain; TSP-1, thrombospondin 1-like domain; vWD, von Willebrand factor type D-like domain. ac, anchor cell; ag, adhesive gland; agb, adhesive gland body; agn, adhesive gland neck; agv, adhesive gland vesicle; ao, adhesive organs; b, brain; cg, cement glands; e, eyes; eg, egg; ep, epidermis; fgo, female genital opening; g, gut; mgo, male genital opening; mo, mouth; o, ovaries; ph, pharynx; rg, releasing gland; rgb, releasing gland body; rgn, releasing gland neck; rgv, releasing gland vesicles; t, testes; tp, tail plate. [Scale bars, 100 µm (*D*) and 20 µm (*E* and *H*).] Schemes modified from ref. 29, which is licensed under CC BY 4.0.

We have identified two large adhesive proteins, *M. lignano* adhesion protein 1 (Mlig-ap1) and Mlig-ap2, comprising 5,407 and 14,794 amino acids, respectively. *Mlig-ap1* and *Mlig-ap2* transcripts were expressed in cells located in the flatworm tail (Fig. 1 *A–G*). Double-fluorescence in situ hybridization confirmed the expression of both genes in the same cells (Fig. 1 *H–J*). A search for the protein sequences of Mlig-ap1 or Mlig-ap2 in the nonredundant database for the National Center for Biotechnology Information (NCBI) revealed no significant homology with any known protein. Mlig-ap1 was characterized by lysine- and arginine-rich regions (KR-regions A to C) (*SI Appendix*, Fig. S1) at the N- and C-terminal ends, together comprising more than 3,600 amino acids. These regions were extremely positively charged, with an isoelectric point of 14.43 (KR-region A), 14.54 (KR-region B), and 14.62 (KR-region C). Several domains were present in the middle portion of Mlig-ap1, including a C-type lectin-binding domain, a von Willebrand factor D domain (which includes a von Willebrand factor domain, a C8 domain with eight conserved cysteines, and a trypsin inhibitor-like domain), and a fibrillin-like region containing a series of 17 EGF-like calcium-binding modules (Fig. 1*K*). The Mlig-ap2 amino acid sequence contained two von Willebrand factor domains, two trypsin inhibitor-like domains, a low-density lipoprotein receptor domain at the N-terminal end, and multiple thrombospondin type 1 repeats at the C-terminal end. The central region of the Mlig-ap2 amino acid sequence was characterized by 46 repeats (Fig. 1*K*) comprising 21 repetitions of a 255-amino acid sequence, followed by 25 repeats of a 221-amino acid sequence (*SI Appendix*, Fig. S2).

Two previous in situ hybridization screens of tail-specific transcripts of *M. lignano* (22, 24) revealed that multiple independent transcripts of the MLRNA815 transcriptome (21) were expressed in the tail (22, 24). Based on the recently published genome of *M. lignano* (23, 25), we here show that six unconnected transcripts of this screen mapped to Mlig-ap1 (*SI Appendix*, Fig. S3) and four were part of Mlig-ap2 (*SI Appendix*, Figs. S4 and S5).

Next, we confirmed that adhesive secreting cells in the flatworm tail contained Mlig-ap1 and Mlig-ap2. In a previous study, we showed that peanut agglutinin-lectin (lectin PNA) was an adhesive gland cell marker (29). However, the nature of the associated protein was not known. Here, we performed lectin PNA pull-down experiments (*n* = 3) and showed that the PNA target sugar Gal-β(1–3)-GalNAc was part of the Mlig-ap2 glycosylation. Multiple peptides of Mlig-ap2 were found by mass spectrometry in each experiment (Dataset S1). Two control pull-downs with PNA preincubated with its inhibitory monosaccharide d-galactose proved the specificity of the pull-down, since no or only one peptide was found in the eluates (Dataset S1). Additional whole-mount staining with PNA compared with control staining with blocked PNA showed that all labeling in the head and tail was gone when PNA was inhibited with d-galactose (*SI Appendix*, Fig. S6) (also see ref. 29). Next, we demonstrated that *Mlig-ap1* was expressed in the adhesive gland cells (*SI Appendix*, Fig. S7 *A*–*C*) but not in anchor cells (12, 29) (*SI Appendix*, Fig. S7 *D*–*F*). We generated multiple Mlig-ap1– and Mlig-ap2–specific antibodies (see *SI Appendix*, Figs. S3 and S4 for the epitope locations) and corroborated their expression in the adhesive gland cells (*SI Appendix*, Fig. S8). In summary, the expression data confirm the localization of both mRNAs and proteins in the adhesive gland cells.

### Adhesive Gland Cells Secrete Mlig-ap1 and Mlig-ap2.

**Fig. 2.**
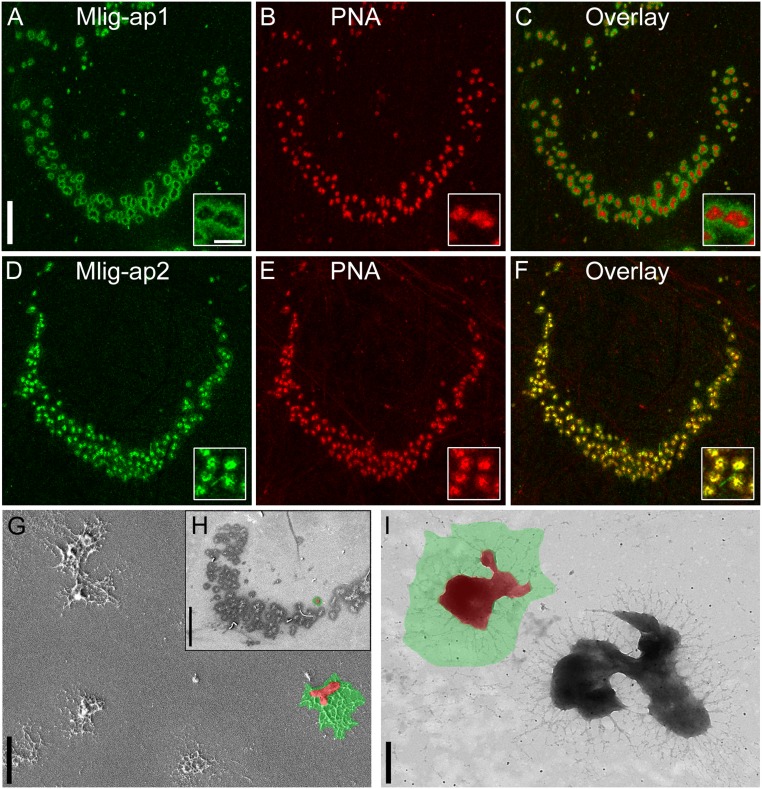
Spatial distribution of Mlig-ap1 and Mlig-ap2 on *M. lignano* footprints. (*A*–*C*) Confocal projection of double labeling of footprint with antibody against Mlig-ap1 and lectin PNA. (*D*–*F*) Confocal projection of double labeling of footprint with antibody against Mlig-ap2 and lectin PNA. (*G* and *H*) Scanning electron microscopy pictures of single adhesive organ footprints in detail (*G*) and a whole tail-plate footprint (*H*), false-colored: Mlig-ap1 is indicated in green, and Mlig-ap2 is in red. (*I*) Transmission electron microscopy picture of two single adhesive organ footprints, false-colored: area of distribution of Mlig-ap1 (green) and Mlig-ap2 (red). For comparison with a footprint lacking Mlig-ap1, see Fig. 5*B*. [Scale bars, 20 µm (*A* and *H*), 2 µm (*G*), and 500 nm (*A*, *Inset* and *I*).]

Next, we explored whether the flatworms secreted these adhesive proteins when they attached to a substrate. During attachment (Movie S1), the flatworms left footprints of Mlig-ap1 and Mlig-ap2 on the surface (Fig. 2). We use the term “footprint” for trace amounts of the secreted material left behind on the surface after detachment. We want to emphasize that the temporary adhesion of *M. lignano* must be seen with respect to the capacity of the animal to detach from the surface while the footprint material remains on the surface. Notably, Mlig-ap1 and Mlig-ap2 exhibited distinct spatial distributions within an imprint of a single adhesive papilla (Fig. 2). Mlig-ap1 was deposited in a ring-like organization at the outer margin of the adhesive footprint (Fig. 2*A* and *SI Appendix*, Fig. S9). In contrast, Mlig-ap2 accumulated mostly in the center of the adhesive papillae (Fig. 2 *D–F*, *Insets*). Scanning electron microscopy and transmission electron microscopy corroborated the shape of the adhesive footprint (Fig. 2 *G–I*). Using the lectin PHA-E that specifically labels the glycocalyx of *M. lignano* (29), we confirmed that glycocalyx was not left on the adhesive footprints (*SI Appendix*, Fig. S10).

The presence of Mlig-ap1 and Mlig-ap2 in the adhesive footprints was further corroborated using mass spectrometry. To maximize the number of peptides available for mass spectrometry, proteases (trypsin, LysC, LysN, chymotrypsin, and V8) were applied in independent experiments together with deglycosylation of proteins. Multiple peptides of both adhesive proteins were identified (*SI Appendix*, Figs. S11 and S12 and Dataset S2). Likewise, the same protein regions were identified in amputated tail samples, which were processed for mass spectrometry (Dataset S3). However, the extremely high lysine and arginine content of Mlig-ap1 hindered the generation of suitable peptides (trypsin cuts at lysine and arginine amino acids) for mass spectrometry (*SI Appendix*, Fig. S11). The protein regions identified by the mass spectrometry of the amputated tails were similar to the peptides identified in the adhesive footprints. Therefore, we conclude that both adhesive proteins were secreted and participated in the attachment.

### RNAi of Mlig-ap1 and Mlig-ap2 Resulted in a Nonadhesion Flatworm Phenotype.

**Fig. 3.**
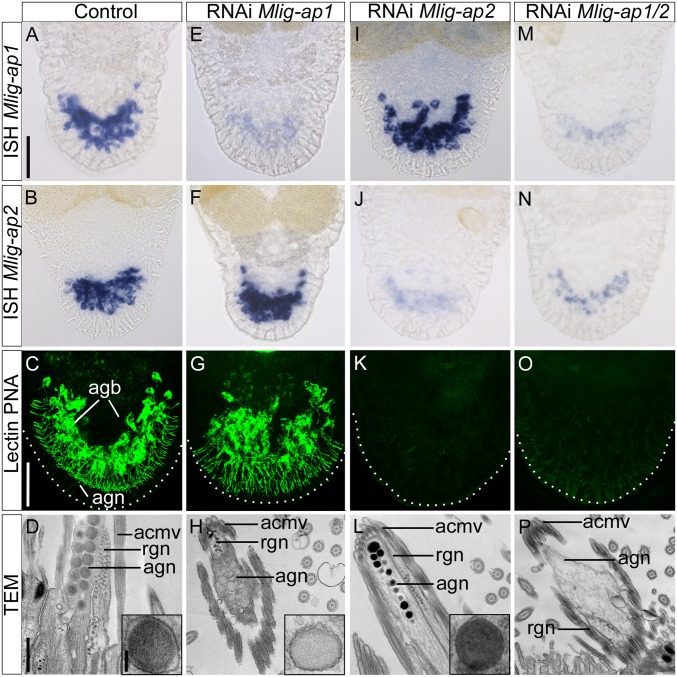
RNAi knockdown of adhesive proteins. (*A*–*D*) Control animals: in situ hybridization of adhesive proteins (*A* and *B*), lectin PNA labeling (confocal projection) (*C*), and ultrastructural analysis with detail of the adhesive vesicle (TEM) (*D*). Note the electron-lucent periphery and the electron-dense core of the vesicle (*D*, *Inset*). (*E*–*H*) *Mlig-ap1* RNAi: in situ hybridization of adhesive proteins (*E* and *F*), lectin PNA labeling (confocal projection) (*G*), and ultrastructural analysis with detail of the adhesive vesicle (TEM) (*H*). Note the lack of the dense core of the vesicle (*H*, *Inset*). (*I*–*L*) *Mlig-ap2* RNAi: in situ hybridization of adhesive proteins (*I* and *J*), lectin PNA labeling (confocal projection) (*K*), and ultrastructural analysis with detail of the adhesive vesicle (TEM) (*L*). Note the lack of the electron-lucent periphery of the vesicle (*L*, *Inset*). (*M*–*P*) Double RNAi: in situ hybridization of adhesive proteins (*M* and *N*), lectin PNA labeling (confocal projection) (*O*), and ultrastructural analysis (TEM). Note the complete absence of adhesive vesicles in the adhesive gland cell (*P*). Dotted lines in confocal images indicate the border of the tail plate. acmv, anchor cell microvilli; agb, adhesive gland body; agn, adhesive gland neck; rgn, releasing gland neck. [Scale bars, 40 µm (*A* and *C*), 500 nm (*D*), and 100 nm (*D*, *Inset*).]

We performed functional analyses by RNAi to assess the involvement of Mlig-ap1 and Mlig-ap2 in *M. lignano* adhesion. We applied RNAi on tail-amputated adults for 9 d until complete tail regeneration was achieved (29, 34) (Fig. 3). In regenerated control animals, *Mlig-ap1* and *Mlig-ap2* expression (Fig. 3 *A* and *B*), PNA labeling (Fig. 3*C*), and the morphology of the adhesive vesicles were normal (Fig. 3*D*) (29) after 9 d of tail regeneration. The control flatworms started to adhere by the third day of regeneration and, after 9 d, the flatworms were attaching 10.5 times per min (*n* = 40, SD 2.9) (Movie S2).

RNAi knockdown of Mlig-ap1 during the 9 d of tail-plate regeneration resulted in a significant reduction of *Mlig-ap1* expression (Fig. 3*E*), while *Mlig-ap2* mRNA levels (Fig. 3*F*) and PNA labeling of Mlig-ap2 (Fig. 3*G*) were not affected. Notably, the dense core of adhesive vesicles in the tail was no longer present (Fig. 3*H*). In the attachment assays, the treated animals were not able to adhere securely compared with the control flatworms. Rather, the RNAi animals experienced a minor delay in their forward movement (Movie S3).

RNAi knockdown of *Mlig-ap2* showed no effect on *Mlig-ap1* expression (Fig. 3*I*) but led to a significant reduction of *Mlig-ap2* mRNA (Fig. 3*J*), a lack of PNA staining in the adhesive glands (Fig. 3*K*), and adhesive vesicles that lacked the electron-lucent periphery and appeared homogeneous (Fig. 3*L*). The knockdown of Mlig-ap2 led to a complete nonadhesive phenotype (*n* = 40) (Movie S4). Notably, no Mlig-ap1 footprints were present after Mlig-ap2 RNAi. These findings suggest that Mlig-ap1 had no or only weak interaction with the surface and that Mlig-ap1 alone was not sufficient for animal attachment. The same results were obtained with a double-RNAi experiment of Mlig-ap1 and Mlig-ap2 (Fig. 3 *M–P* and Movie S5). At the ultrastructural level, double knockdown resulted in a complete loss of adhesive vesicles (Fig. 3*P*). Furthermore, RNAi performed with dsRNA against the middle region of the *Mlig-ap1* or *Mlig-ap2* mRNA combined with in situ hybridization staining with different probes located at the 3′ and 5′ regions showed an effective knockdown in all cases (*SI Appendix*, Fig. S13). In summary, RNAi experiments confirmed that both proteins exhibit a spatially restricted distribution within the adhesive vesicles and that they play an essential role in *M. lignano* adhesion.

### The Attachment and Detachment Process.

We tested various conditions that might interfere with the *M. lignano* attachment or release process (*SI Appendix*, Table S1). First, we wanted to exhaust the flatworm’s attachment capacity by manually pipetting or by keeping the flatworms on a horizontal shaker for up to 24 h. We found no reduction in attachment capacity. Instead, the animal sequentially used different adhesive organs (Movie S6). We hypothesize that they recharged the tip of the gland cell necks with new adhesive vesicles. Accordingly, the ultrastructure of the adhesive gland cell necks of animals that were manually pipetted for 5 min showed a significantly reduced number of adhesive vesicles in some of the adhesive gland cells (*SI Appendix*, Fig. S14).

Next, we changed the parameters of the culture medium with respect to salinity (2 to 60 ppm), pH (pH 4 to 10), and temperature (10 to 32 °C). None of these changes disrupted the flatworms’ ability to attach and release (*SI Appendix*, Fig. S15).

**Fig. 4.**
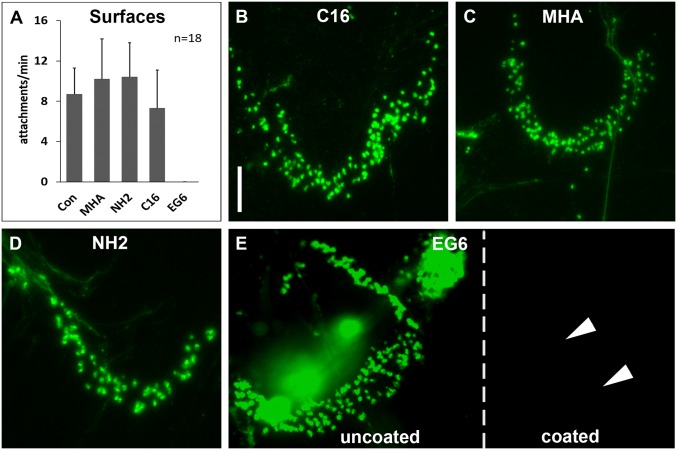
Attachment on different self-assembled monolayers. (*A*) Average number of attachments per min on glass (Con), anionic (MHA), cationic (NH2), hydrophobic (C16), and neutral hydrophilic (EG6) surfaces. (*B*–*E*) Fluorescence images of footprints stained with PNA on different SAMs: (*B*) C16 (hydrophobic), (*C*) MHA (anionic), (*D*) NH2 (cationic), and (*E*) EG6 (neutral hydrophilic). Note that on EG6, footprints can only be found on the uncoated side, while no footprints are visible on the coated area (indicated with white arrowheads). The dashed white line indicates the border between hydrophilic coating and uncoated regions of the slide (*E*). Error bars indicate SD (*n* = 18). (Scale bar, 25 µm.)

We next evaluated flatworm attachment onto different surfaces. We put the flatworms onto glass, plastic, wood, leaves, stone, and metal. In all experiments, regular attachment and release were observed. Self-assembled monolayers (SAMs) have been used frequently as model surfaces in bioadhesion and biofouling research, for example to test the attachment of bacteria, algal spores, and proteins (35–39). We tested SAMs with anionic, cationic, hydrophobic, or neutral hydrophilic properties. The flatworms attached well to anionic, cationic, and hydrophobic surfaces, but they did not attach to the surface with the neutral hydrophilic coating (Fig. 4*A*). These observations corresponded to the results of the adhesive footprint labeling. While footprints were present on the anionic, cationic, and hydrophobic surfaces (Fig. 4 *B–D*), no footprints were found on the neutral hydrophilic surface (Fig. 4*E*).

We then tested surfaces with gradually reduced hydration. We use the term “hydration” for hydrophilic surfaces with different levels of internal hydration. We generated oligo(ethylene glycol)-terminated alkanethiol-coated surfaces with six, four, two, and one ethylene glycol units (EG6, EG4, EG2, and EG1). While the contact angles of the EG1 to 6 surfaces were comparable, the internal hydration of the EGx chains was different (see *Material and Methods* for details on contact angle and hydration of the EG1 to 6 SAMs). The flatworms could not attach to the EG6, EG4, and EG2 surfaces. However, the flatworms experienced a delay in their forward movement when they tried to attach to the EG1-coated surface. No adhesive footprints were found on the EG6, EG4, and EG2 surfaces, while footprints were present on the EG1-coated slides (*SI Appendix*, Fig. S16). These results indicated that flatworm adhesion was not possible on highly hydrated surfaces. We expect that on regular surfaces, Mlig-ap2 displaced water to directly interact with the substrate surface for attachment. On highly hydrated surfaces, however, the water molecules cannot be displaced, and the interaction of Mlig-ap2 with the surface was impeded. Direct demonstration of the displacement of interfacial water during attachment is a considerable experimental challenge and currently not feasible due to spatial and temporal limits of available methods.

**Fig. 5.**
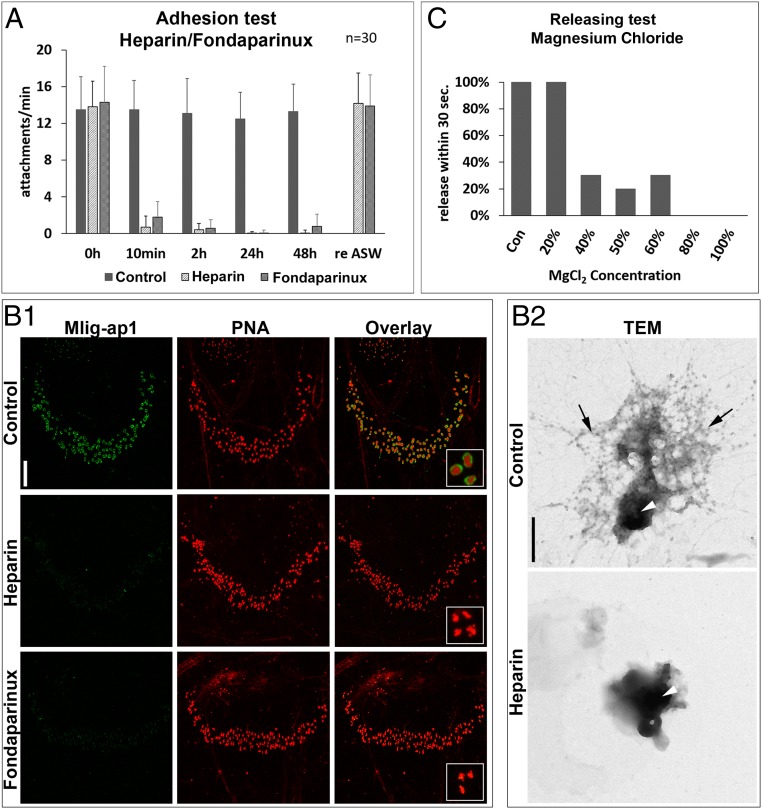
Interference of attachment and detachment. (*A*) Average number of attachments with 2% heparin or 10 mg/mL Fondaparinux in ASW over time. (*B*1 and *B*2) Change of footprint appearance under the influence of heparin. (*B*1) Confocal projection of double labeling of footprints with Mlig-ap1 antibody (green) and lectin PNA under normal culture conditions (control; *Upper*), 2% heparin (*Middle*), and 10 mg/mL Fondaparinux (*Lower*). Note that footprints were no longer Mlig-ap1–immunopositive with heparin or Fondaparinux added to the ASW. (*B*2) TEM of a single adhesive organ footprint under normal culture conditions (control; *Upper*) and 2% heparin (*Lower*). Black arrows indicate presumed Mlig-ap1 distribution, and white arrowheads indicate that of Mlig-ap2. (*C*) Total percentage of worms that were able to release within 30 s in different MgCl_2_ (isoosmolar to ASW, 7.14%) concentrations. Error bars indicate SD (*n* = 30). [Scale bars, 20 µm (*B*1) and 500 nm (*B*2).]

We next investigated whether carbohydrates played a role in the attachment or release process. Remarkably, 2% heparin in artificial sea water (ASW) completely inhibited attachment (Fig. 5*A*). However, the typical attachment behavior (movement of the tail plate) did not change with the presence of heparin even if no bonding with the surface occurred. The flatworms immediately regained their attachment capabilities if they were transferred to normal ASW (Fig. 5*A*). Selectively desulfated heparin oligosaccharides and chondroitin sulfate also impeded flatworm attachment (*SI Appendix*, Fig. S17*A*). Various concentrations of glucose, galactose, or lectin PNA had no effect on flatworm attachment and release (*SI Appendix*, Fig. S17*B* and Table S1). Notably, only Mlig-ap2 footprints were found, while Mlig-ap1 was missing (Fig. 5*B*, 1). Likewise, the presence of heparin altered the morphology of the footprint. Fibrous material surrounding the dense center was missing (Fig. 5*B*, 2). The fibrous periphery of the footprint (Figs. 2*I* and 5*B*, 1) presumably consisted of Mlig-ap1, while the dense center was composed of Mlig-ap2. In nature, heparin binds to antithrombin III through a specific sulfated pentasaccharide sequence. An equivalent synthetic pentasaccharide known as Fondaparinux (GlaxoSmithKline) is used as an anticoagulant medication. As with the heparin experiments, the flatworms could not attach to the substrate with 10 mg/mL of Fondaparinux in the culture medium (Fig. 5*A*). Likewise, the adhesive footprints were only composed of Mlig-ap2 (Fig. 5*B*, 1). However, treatment of regular adhesive footprints with heparin or Fondaparinux did not affect the Mlig-ap1 and Mlig-ap2 localization (*SI Appendix*, Fig. S18). These observations demonstrated that negatively charged molecules impair flatworm attachment. From those observations, we propose that heparin or Fondaparinux adsorbed to the positively charged Mlig-ap1 when it was released from the vesicles, preventing the cohesive function of Mlig-ap1. We predict that a small negatively charged molecule served as a releasing factor in *M. lignano*.

Next, we replaced the culture medium (ASW) with variable concentrations of isoosmolar magnesium chloride (7.14% MgCl_2_ in H_2_O). The flatworms were able to attach at all concentrations. However, they were unable to detach from 80 to 100% of 7.14% MgCl_2_ (Fig. 5*C*) despite strong movements (Movie S7). In contrast, all worms in regular ASW detached within 30 s. In a separate experiment, the presence of 1-phenoxy-2-propanol (0.1% in ASW), a common muscle relaxant, inhibited muscle contraction but did not alter the adhesion or releasing process (Movie S8). This finding suggests that muscle contraction was not involved in *M. lignano* attachment or release. Next, we assumed that the presence of positively charged ions might affect the releasing process. To further evaluate this hypothesis, we tested l-lysine– and arginine-monohydrochloride solutions (10% in ASW). The flatworms moved and behaved normally, but once they attached to the substrate, 70% (*n* = 10) of the animals could not release (Movie S9). When the positively charged peptide used for antibody production (SRKPRRKNRKSRKP) was added to the culture medium (4 mg/100 µL), detachment was also impaired. These findings indicate that positively charged molecules interfere with the flatworm detachment process.

### A Model for Temporary Adhesion.

**Fig. 6.**
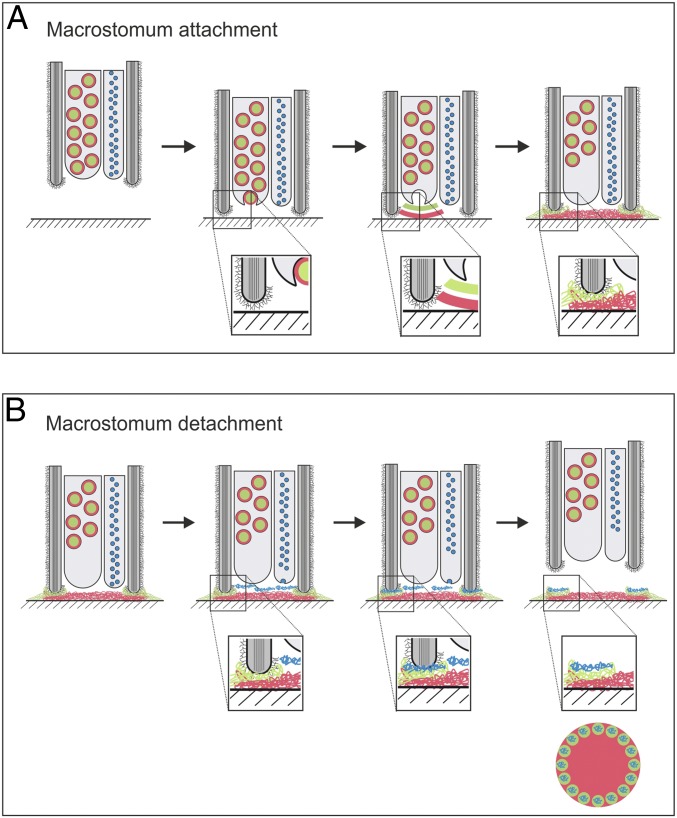
Model for temporary adhesion in the flatworm *M. lignano*. Schematic illustration of attachment and release of a single adhesive organ in *M. lignano*. The scheme depicts a sagittal section through the tip of an adhesive organ during attachment (*A*) and release (*B*). (*A*) Natural attachment under normal culture conditions. (*B*) Natural release under normal culture conditions. See text for details. Color code: Mlig-ap1, green; Mlig-ap2, red; releasing factor, blue; microvilli (gray) with glycocalyx.

We propose a data-based model for temporary adhesion based on *M. lignano* attachment and release (Fig. 6*A*). When the flatworm’s adhesive organs contacted a surface, the adhesive proteins Mlig-ap1 and Mlig-ap2 were secreted. Mlig-ap2, the adhesion protein, was assumed to displace water molecules and attached to the surface. Mlig-ap1, the cohesion protein, connected Mlig-ap2 to the microvilli of the anchor cell. Upon detachment (Fig. 6*B*), the flatworms discharged the releasing vesicles. We suggest that a small negatively charged molecule associated with the positively charged Mlig-ap1 and induced detachment.

**Fig. 7.**
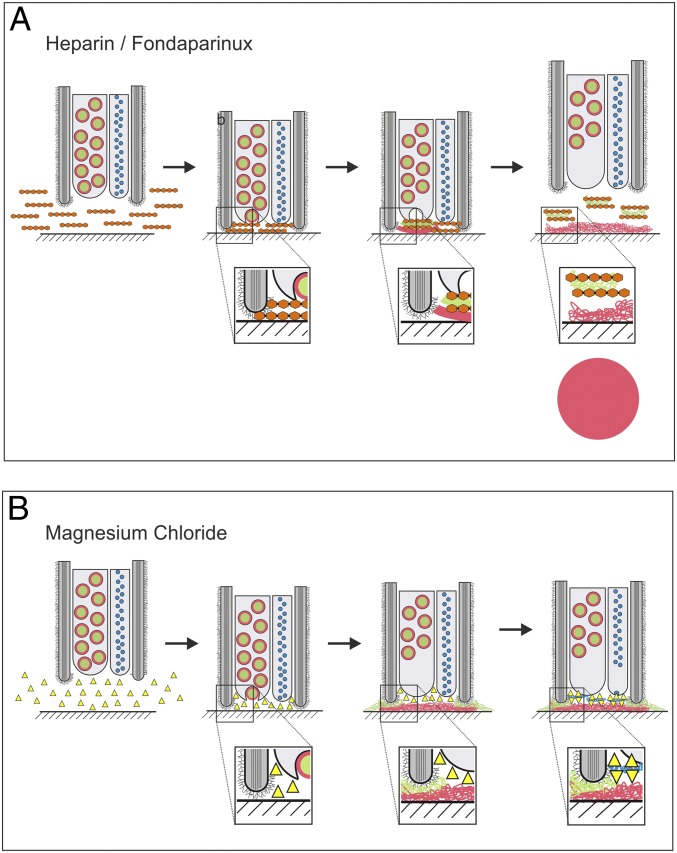
Model for temporary adhesion in the flatworm *M. lignano*. Inhibition of attachment and release. Schematic illustration of a sagittal section through the tip of an adhesive organ during inhibition of attachment with heparin/Fondaparinux (*A*) and inhibition of release with positively charged molecules like MgCl_2_ or lysine/arginine (*B*). Color code: Mlig-ap1, green; Mlig-ap2, red; releasing factor, blue; heparin, orange; positively charged molecules, yellow; microvilli (gray) with glycocalyx.

This model is supported by the observations that negatively charged carbohydrates bound to the positively charged lysine and arginine repeats of Mlig-ap1, inhibited its cohesive function, and, consequently, the attachment (Fig. 7*A*). In contrast, the presence of positively charged molecules (lysine, arginine, or the SRKPRRKNRKSRKP peptide) or ions (MgCl_2_) in the medium hindered flatworm detachment (Fig. 7*B*). Additionally, electron microscopic staining methods revealed the presence of periodic acid-Schiff–positive polysaccharides (*SI Appendix*, Fig. S19*A*), and element analysis revealed a low density of proteins in the releasing vesicles (*SI Appendix*, Fig. S19*B*).

## Discussion

Understanding the mechanisms involved in biological adhesion is important for interfacial chemistry, biofouling, and biomedicine, including the development of novel biomimetic glues. An increased knowledge of the fundamental mechanisms of bioadhesion and the related macromolecules is crucial for the development of next-generation adhesives that can rapidly adhere in wet environments and have a reversible adhesive capacity.

We studied the temporary adhesion of the flatworm *M. lignano*. We identified only two adhesive proteins in *M. lignano*, and no other candidates have been found in previous studies (22, 24) or in the current work. In comparison, six foot proteins have been identified in mussel byssi (40), five cement proteins have been described in barnacles (41), and five cement proteins, one of which (c3) occurs in at least two variations, are present in tubeworms (42, 43). Proteomic and transcriptomic studies in sea stars found up to 34 potential adhesive proteins (15). A quantitative proteomic investigation of the adhesive secretions of the sea urchin *Paracentrotus lividus* revealed 163 potential adhesion candidates (16). In *Hydra*, 21 disk-specific adhesive candidate proteins were identified (44). Therefore, most species possess multiple adhesive proteins, and the exact number of adhesive proteins in many species has yet to be elucidated.

The only two previously described proteins in temporary adhesion are the sea star foot protein 1 (Sfp1) (17) and the sea urchin nectin (18). Sfp1 shares many characteristics with Mlig-ap1 regarding carbohydrate- and metal-binding domains such as von Willebrand type D domains, galactose-binding lectin domains, C8 domains, and one calcium-binding EGF-like domain (17). Sfp1 has been described as the main cohesive protein for the sea star *Asterias rubens*. Another common feature of Sfp1 and Mlig-ap1 is a high cysteine content. Sfp1 reportedly has a cysteine content of 5% (17), while the domain region of Mlig-ap1 from amino acid 2758 to 4310 displays a cysteine content of 11%. A high proportion of cysteines can also be found in the mussel foot protein 2 (mfp-2) and the barnacle cement protein 20k (cp-20k). Interestingly, mfp-2, like Mlig-ap1,was shown to contain a number of EGF repeats, which play an important role in cohesion (40).

The role of glycosylation in bioadhesion is not well-understood, even though it is commonly found in marine bioadhesives. We have confirmed that the sugar Gal-β(1–3)-GalNAc is associated with Mlig-ap2. Glycosylation has been shown to be involved in the adhesion of limpets, mussels, barnacles, algae, and sea stars (45–49). These correlations indicate that certain protein domains could be common in cohesive proteins, while glycosylation is prevalent in adhesive proteins.

So far, the releasing mechanism of temporary adhesion and the substances involved in detachment have been poorly understood. Tyler (13, 14) showed that only the large adhesive vesicles of adhesive gland cells are expelled during attachment. Furthermore, Tyler postulated that detachment was caused by the secretion of a chemical substance (13, 14). Hermans (50) suggested a duo-gland adhesive system for echinoderm tube feet. Hermans predicted that the deadhesive substance comprised glycosaminoglycans that outcompeted the binding of the adhesive material to the glycocalyx. Hermans’s proposed model is supported by the observation that heparin inhibits adhesion in the sea star *Leptasterias hexactis* (51).

Another proposed detachment model involves the secretion of cleaving enzymes to break the bond between the animal and the adhesive material (7, 15, 52–54). In the proteome of *A. rubens* footprints, two proteases have been identified, while in the tube feet of the sea urchin *P. lividus*, several proteases and glycosylases are expressed (15, 16). Nevertheless, it is unknown if these proteases are expressed in deadhesive gland cells and if they indeed play a role during detachment. In *M. lignano*, no proteases playing a role in detachment have been identified in adhesive organs (22, 24). Furthermore, in the enzymatic detachment theory, it has been proposed that parts of the glycocalyx might remain incorporated into the footprints (7, 52). The *M. lignano* footprints were not labeled by the lectins PHA-E and PHA-L (*SI Appendix*, Fig. S10), which strongly reacted to the flatworm’s glycocalyx (29). Based on our findings, we assume that enzymatic and muscular detachment is unlikely in *M. lignano*. In addition, in video microscopy, no muscle contraction was observed during detachment. We propose that detachment of *M. lignano* is based on the release of negatively charged substances that react with Mlig-ap1.

The generation of chemically defined and well-characterized surfaces in the form of self-assembled monolayers (55) allowed us to study the impact of different hydration levels of the surface on the attachment of animals. Notably, attachment of *M. lignano* onto strongly hydrated surfaces (EG6, EG4, EG2) was not possible, while footprints could be detected on the less hydrated EG1 SAM. Previous experiments (39, 56–58) show an inferior fouling resistance of EG1, compared with EG2 to 6, and modeling (59, 60) demonstrates that this is related to the weaker hydration of the EG1 segment. Our results are in complete coherence with this literature, which supports the view that strong hydration of the surfaces prevents flatworm attachment (see also comments on EGx properties in *Materials and Methods*). A negative correlation between wettability and attachment was observed for algal spores, diatoms, bacteria, and fibrinogen (35, 61–63). Our data suggest that a low-level surface hydration is critical for *M. lignano* attachment. However, we have no direct evidence for the displacement of water by Mlig-ap2. The presence of a water layer on strongly hydrated surfaces impedes interaction of Mlig-ap2 with the surface and inhibits animal attachment.

Currently available medical adhesives, such as cyanoacrylates, gelatin resorcinol, and fibrin-based glues, have several limitations. They often have cytotoxic effects (64) or possess only weak cohesive strength (65). Bioinspired adhesives could feature strong attachment forces under wet conditions and provide nontoxic and biodegradable alternatives (4, 66). Elucidation of the reversible adhesion of flatworms and other organisms may inspire the development of new adhesives suitable for medical applications.

## Materials and Methods

### Animal Cultures.

*M. lignano* (28) cultures of the inbred line DV1 (27) were kept as described (67).

### Mlig-ap1 and Mlig-ap2 Characterization.

A differential transcriptome approach (21) and two in situ hybridization screens (22, 24) revealed 10 transcripts of the MLRNA110815 transcriptome (21) to be expressed in the adhesive organs. With the advent of the *M. lignano* genome (23, 25), it became evident that six transcripts (RNA815_13121.1, RNA815_13121.2, RNA815_13121.3, RNA815_317.1, RNA815_27695.1, RNA815_27695.2) were part of Mlig-ap1 (*SI Appendix*, Fig. S3) and four (RNA815_16005, RNA815_300, RNA815_21583, RNA815_23142) belonged to Mlig-ap2 (*SI Appendix*, Fig. S4). Full-length Mlig-ap1 is part of the genomic contig scaf1170 of the Mlig_3_7 genome assembly (25). The final Mlig-ap1 sequence (deposited in GenBank; accession no. MH586844) (68) was manually curated using the following sequence data: the genomic contig scaf1170, six transcripts of the MLRNA110815 transcriptome (see above), and cloned and Sanger sequenced regions within Mlig-ap1 (*SI Appendix*, Fig. S3). Full-length Mlig-ap2 is part of the genomic contig scaf3366 of the Mlig_3_7 genome assembly (25). The final Mlig-ap2 sequence (deposited in GenBank; accession no. MH586845) (68) was manually curated using the following sequence data: the genomic contig scaf3366, four transcripts of the MLRNA110815 transcriptome (see above), and cloned and Sanger sequenced regions within Mlig-ap2 (*SI Appendix*, Fig. S4). We corroborated the expression patterns and confirm that all transcripts show expression in the tail (*SI Appendix*, Fig. S5). The isoelectric point of Mlig-ap1 was determined using Isoelectric Point Calculator software (69). Conserved protein domain searches were performed using InterPro software (InterPro 67.0) (70) and NCBI conserved domain search (71).

### Gene Isolation.

Sequences from the transcriptomes MLRNA110815 (21), MLRNA131024 (www.macgenome.org), and Mlig_RNA_3_7_DV.v1 (25) and genomes ML2 (23) and Mlig_3_7 (25) were used for primer design (*SI Appendix*, Table S2). Obtained fragments were cloned in pGEM-T (Promega) and sequenced by Microsynth.

### Whole-Mount in Situ Hybridization and Double-Fluorescence in Situ Hybridization.

Whole-mount in situ hybridization (WISH) was done according to the protocol published previously (12). Double-fluorescence in situ hybridization (dFISH) was performed adapting the WISH protocol using the TSA signal amplification system (PerkinElmer). In short, additional to the DIG-labeled ISH probe, a second fluorescein-labeled probe was generated using fluorescein labeling mix (Roche). Probe concentration was reduced to 0.02 ng/µL for both probes. Anti–fluorescein-HRP (PerkinElmer) and anti–DIG-HRP (PerkinElmer) were diluted 1:500. Signal amplification was done with amplification diluent fluorescein-tyramide and amplification diluent Cy3-tyramide (PerkinElmer) for 4 min each. Samples were mounted in Vectashield (Vector Laboratories) and pictures were taken with a Leica SP5 II confocal scanning microscope. Stacks were acquired sequentially and *z*-projected.

### Mass Spectrometry Sample Preparation.

Proteins from worm footprints or worm tissue or pull-down experiments were reduced with 50 µL 10 mM DTT at 56 °C for 30 min and alkylated with 50 µL 55 mM iodoacetamide at room temperature for 20 min. Samples were digested either with trypsin, chymotrypsin, peptidase V8, lysine C, or lysine N at 37 °C overnight. The tryptic peptides were purified by ZipTip C18 pipette tips (Millipore) according to the manufacturer’s instructions before nano-LC-electrospray ionisation-MS analysis. Samples were analyzed using an UltiMate 3000 nano-HPLC system coupled to a Q Exactive mass spectrometer (both Thermo Scientific). Peptides were separated on a homemade fritless fused-silica microcapillary column (75 μm i.d. × 280 μm o.d. × 10 cm length) packed with 3-μm reversed-phase C18 material (Reprosil). Solvents for HPLC were 0.1% formic acid (solvent A) and 0.1% formic acid in 85% acetonitrile (solvent B).

### Mass Spectrometry Data Analysis.

Data analysis was performed using Proteome Discoverer software (Thermo Scientific) with the search engine Squest against the translated MLRNA110815 and MLRNA131024 transcriptomes containing complete sequence of Mlig-ap1 and Mlig-ap2, while single transcripts belonging to the two proteins were manually deleted. Precursor and fragment mass tolerance was set to 10 ppm and 0.02 Da, respectively, and up to two missed cleavages were allowed. Carbamidomethylation of cysteine and oxidation of methionine were set as variable modifications. Peptide identifications were filtered at 1 or 5% false discovery rate. Data have been deposited to the ProteomeXchange Consortium (72) via the PRIDE (73) partner repository (dataset identifier PXD012626).

### RNA Interference.

RNAi was done as previously described (12, 32). Negative control animals were treated with *Luciferase* dsRNA or without the presence of dsRNA. As shown in previous studies (26, 30), *Luciferase* dsRNA controls did not show any mock effect of the treatment and did not result in any morphological change in various tissues of the animals (32). After 9 d of regeneration, phenotypes were observed in vivo by performing attachment assays (see below). The efficacy of the knockdown was verified by video microscopy, WISH, lectin PNA staining, or transmission electron microscopy (TEM).

### Lectin Pull-Down.

Lectin pull-down was done using Thermo Scientific Pierce streptavidin magnetic beads (88817). Briefly, protein from 1,000 tails was isolated and precleaned with 50 µL streptavidin magnetic beads. The precleaned protein was incubated with biotinylated PNA overnight at 4 °C. Then, the protein was incubated with streptavidin magnetic beads for 2 h at room temperature, magnetic beads were washed three times with Tris-buffered saline (TBS) (with 5 mM CaCl_2_), and the protein was eluted with 5% formic acid. For control pull-down experiments, the PNA was preincubated with its inhibitory monosaccharide d-galactose (0.4 M) for 2 h at 4 °C. Identified peptides are listed in *SI Appendix*, Table S1.

### Lectin PNA and Antibody Labeling of Whole-Mount Animals.

Anti–Mlig-ap1 and anti–Mlig-ap2 antibodies were raised in rabbits by GenScript. For Mlig-ap1, antibodies were produced against the following peptides: SRKPRRKNRKSRKP (antibody name, AP1_R2; total abundance of peptide sequence, 56×) and KNNKRRVRKARKNN (antibody name, AP1_R4; total abundance of peptide sequence, 25×). For anti–Mlig-ap2, antibodies against the following peptides were used: TSRKSTGRTTRQKK (antibody name, AP2_R2; total occurrence of peptide sequence, 21×) and VNRETPKEPKKALS (antibody name, AP2_R3; total occurrence of peptide sequence, 26×). A double labeling of adhesive gland cells with lectin PNA and polyclonal antibodies against Mlig-ap1 or Mlig-ap2 was done as described (29). Mlig-ap1 or Mlig-ap2 antibodies were diluted 1:1,000. Controls for PNA labeling were performed by preincubating the lectin with its inhibitory monosaccharide d-galactose (0.4 M) for 2 h at 4 °C.

### Lectin PNA and Antibody Labeling of Footprints.

For footprint labeling, the above-mentioned antibodies and a third polyclonal antibody for Mlig-ap1 were used. It was generated according to the transcript sequence MLRNA110815_13121.1 against the peptide CRKSRKVNEEAWPRSP (antibody name, AP1_R1; total abundance of KSRKVN, 84×). This antibody did successfully stain Mlig-ap1 on the footprints but did not work in whole mounts.

For footprint staining, approximately 20 worms were pipetted on a microscopy glass slide in a drop of ASW and left to adhere for 1 h at room temperature. Specimens were removed and footprints were fixed with 4% paraformaldehyde (PFA) for 30 min at room temperature and processed for antibody or lectin staining.

### Transmission Electron Microscopy.

Chemical fixation and high-pressure freezing were performed as described (71). For TEM visualization of footprints, they were collected on pioloform-coated gold grids. Animals were allowed to attach on the film for 10 min. Footprints were fixed in 4% PFA in PBS and washed five times with TBS followed by five washes in ddH_2_O. Footprints were stained with lead citrate for 30 s and rinsed with ddH_2_O. Images were taken with a Zeiss Libra 120 and iTEM software (Olympus).

Electron spectroscopic imaging and electron energy loss spectroscopy were performed as described (44).

### Periodic Acid-Schiff Cytochemistry at EM Level.

Polysaccharides with vicinal hydroxyl groups were detected with periodic acid-thiocarbohydrazide-silverproteinate according to ref. 75 on 90-nm-thin sections of high-pressure frozen and freeze-substituted EMBed812-embedded animals (74).

### Scanning Electron Microscopy.

Animals were allowed to adhere on a glass slide for about 15 min. Footprints were fixed chemically with 2.5% glutaraldehyde in 0.1 M cacodylate buffer containing 10% sucrose for 1 h. After several rinses with buffer, samples were postfixed with 1% osmium tetroxide in 0.05 M cacodylate buffer for 1 h. After washing, samples were dehydrated in methanol, critical point-dried (Pelco), and sputter-coated with 20-nm gold (CCU-010; Safematic). Samples were examined with a Jeol JSM-7610F field emission scanning electron microscope at 5 to 15 kV.

### Self-Assembled Monolayers.

SAMs were prepared on Si(100) wafers (Topsil) cut into 15-mm × 20-mm pieces which were TL1-cleaned (in a 1:1:5 solution of 25% NH_3_, 30% H_2_O_2_, and 18.2 MΩ⋅cm Milli-Q water, for 10 min at 80 °C) before use. Si pieces were gold-coated in an electron-beam evaporation system with a base pressure of <2 × 10^−8^ mbar. A 20-Å Ti adhesion layer preceded a 2,000-Å Au layer, deposited at a rate of 1 and 5 Å/s, respectively. Thiols used to form SAMs, namely HS(CH_2_)_15_CH_3_ (1-hexadecanethiol; C16) (Fluka), HS(CH_2_)_15_COOH (16-mercaptohexadecanoic acid; MHA) (Sigma-Aldrich), and HS(CH_2_)_16_NH_2_ (16-amino-1-hexadecanethiol; NH2) (ProChimia), were used as received. The synthesis of HS(CH_2_)_15_CONH(CH_2_CH_2_O)_*n*_H, *n* = 1, 2, 4, 6, has been described previously (76). The gold-coated substrates were TL1-cleaned immediately before immersion in thiol solutions, and then incubated in 50 µM thiol solutions in ethanol (99.5%; Solveco) for at least 24 h in the dark. MHA was incubated with 10% (vol/vol) glacial acetic acid in the solution, and the solution of the amine-terminated thiol was adjusted to pH 12 with concentrated NaOH immediately before incubation. After incubation, the surfaces were rinsed with ethanol, ultrasonicated in ethanol for 2 min to remove any physisorbed layers, and dried under a stream of nitrogen gas. The resulting SAMs were evaluated by ellipsometry (*SI Appendix*, Table S3) and contact angle measurements (*SI Appendix*, Table S3). Samples were packed under nitrogen, shipped by overnight courier from Linköping to Innsbruck, and kept in the dark at room temperature until used.

Contact angle data for the SAMs are provided in *SI Appendix*, Table S3. The weak trend of increasing advancing contact angle with longer oligo (ethylene glycol) units in the EGx SAMs is in agreement with previously published data on similar series (39, 76, 77).

While all EGx surfaces are hydrophilic, there is a wealth of information indicating that there are important differences in the internal hydration of the EGx chains. For example, the differences in resistance to biofouling of EGx SAMs have been established in several experiments (39, 56), and early work from Whitesides and coworkers (56, 78) demonstrated that EGx-terminated SAMs with *x* >2 effectively resist the adsorption of proteins. The fouling resistance for these monolayers does not correlate directly with wettability or interfacial free energy, but the structural details of the surfaces are important. Both experiments (57, 58) and modeling (59, 60) by Grunze and coworkers show that the conformations of the EGx segments that were most inert to fouling were those which interact most strongly with water and, in particular, those conformers which allow water molecules to bridge hydrogen-bond acceptor oxygens along the EGx chain. The single ethylene oxide residue in EG1-terminated SAMs does not have the capacity to form such stable bonds with water, and for EG2 this capacity is limited, whereas EG3 has several stable hydrated conformers (59), allowing the polyether chain to coordinate water strongly, and thereby preventing adhesion of approaching molecules.

### Adhesion and Releasing Test Assays.

For adhesion assays, animals were incubated in the respective solutions and left for adaptation for 5 min. A single worm was pipetted on a microscopy slide in a small drop of medium and the attachments per min were counted. For surface tests, single worms were pipetted on the respective surface and the attachments per min were counted. Deposited footprints were processed for lectin or antibody staining. For releasing tests, single animals were put into the solution and the percentage of attached worms able to release within 30 s was evaluated. Movies of live animals were made using a Leica MZ16 F.

## Supplementary Material

Supplementary File

Supplementary File

Supplementary File

Supplementary File

Supplementary File

Supplementary File

Supplementary File

Supplementary File

Supplementary File

Supplementary File

Supplementary File

Supplementary File

Supplementary File

## References

[1] Bianco-Peled H, Davidovich-Pinhas M (2015). Bioadhesion and Biomimetics: From Nature to Applications.

[2] Kord Forooshani P, Lee BP (2017). Recent approaches in designing bioadhesive materials inspired by mussel adhesive protein. J Polym Sci A Polym Chem.

[3] von Byern J, Grunwald I (2010). Biological Adhesive Systems: From Nature to Technical and Medical Application.

[4] Cha HJ, Hwang DS, Lim S (2008). Development of bioadhesives from marine mussels. Biotechnol J.

[5] Waite JH (1983). Adhesion in byssally attached bivalves. Biol Rev Camb Philos Soc.

[6] Gorb SN (2008). Biological attachment devices: Exploring nature’s diversity for biomimetics. Philos Trans A Math Phys Eng Sci.

[7] Flammang P (1996). Adhesion in echinoderms. Echinoderm Stud.

[8] Kamino K (2013). Mini-review: Barnacle adhesives and adhesion. Biofouling.

[9] Stewart RJ, Wang CS, Song IT, Jones JP (2017). The role of coacervation and phase transitions in the sandcastle worm adhesive system. Adv Colloid Interface Sci.

[10] Waite JH (2017). Mussel adhesion—Essential footwork. J Exp Biol.

[11] Santos R (2009). First insights into the biochemistry of tube foot adhesive from the sea urchin *Paracentrotus lividus* (Echinoidea, echinodermata). Mar Biotechnol (NY).

[12] Lengerer B (2014). Biological adhesion of the flatworm *Macrostomum lignano* relies on a duo-gland system and is mediated by a cell type-specific intermediate filament protein. Front Zool.

[13] Tyler S (1976). Comparative ultrastructure of adhesive systems in turbellaria. Zoomorphologie.

[14] Tyler S (1977). Ultrastructure and systematics: An example from turbellarian adhesive organs. Mikrofauna Meeresbodens.

[15] Hennebert E, Leroy B, Wattiez R, Ladurner P (2015). An integrated transcriptomic and proteomic analysis of sea star epidermal secretions identifies proteins involved in defense and adhesion. J Proteomics.

[16] Lebesgue N (2016). Deciphering the molecular mechanisms underlying sea urchin reversible adhesion: A quantitative proteomics approach. J Proteomics.

[17] Hennebert E (2014). Sea star tenacity mediated by a protein that fragments, then aggregates. Proc Natl Acad Sci USA.

[18] Toubarro D (2016). Cloning, characterization, and expression levels of the nectin gene from the tube feet of the sea urchin *Paracentrotus lividus*. Mar Biotechnol (NY).

[19] Laumer CE, Hejnol A, Giribet G (2015). Nuclear genomic signals of the ‘microturbellarian’ roots of platyhelminth evolutionary innovation. eLife.

[20] Egger B, Ladurner P, Nimeth K, Gschwentner R, Rieger R (2006). The regeneration capacity of the flatworm *Macrostomum lignano*—On repeated regeneration, rejuvenation, and the minimal size needed for regeneration. Dev Genes Evol.

[21] Arbore R (2015). Positional RNA-seq identifies candidate genes for phenotypic engineering of sexual traits. Front Zool.

[22] Lengerer B (2018). Organ specific gene expression in the regenerating tail of *Macrostomum lignano*. Dev Biol.

[23] Wasik K (2015). Genome and transcriptome of the regeneration-competent flatworm, *Macrostomum lignano*. Proc Natl Acad Sci USA.

[24] Weber M (2018). A targeted in situ hybridization screen identifies putative seminal fluid proteins in a simultaneously hermaphroditic flatworm. BMC Evol Biol.

[25] Wudarski J (2017). Efficient transgenesis and annotated genome sequence of the regenerative flatworm model *Macrostomum lignano*. Nat Commun.

[26] De Mulder K (2009). Stem cells are differentially regulated during development, regeneration and homeostasis in flatworms. Dev Biol.

[27] Janicke T (2013). Sex allocation adjustment to mating group size in a simultaneous hermaphrodite. Evolution.

[28] Ladurner P, Scharer L, Salvenmoser W, Rieger RM (2005). A new model organism among the lower Bilateria and the use of digital microscopy in taxonomy of meiobenthic Platyhelminthes: *Macrostomum lignano*, n. sp (Rhabditophora, Macrostomorpha). J Zoological Syst Evol Res.

[29] Lengerer B, Hennebert E, Flammang P, Salvenmoser W, Ladurner P (2016). Adhesive organ regeneration in *Macrostomum lignano*. BMC Dev Biol.

[30] Pfister D (2008). Flatworm stem cells and the germ line: Developmental and evolutionary implications of macvasa expression in *Macrostomum lignano*. Dev Biol.

[31] Grudniewska M (2016). Transcriptional signatures of somatic neoblasts and germline cells in *Macrostomum lignano*. eLife.

[32] Kuales G (2011). Boule-like genes regulate male and female gametogenesis in the flatworm *Macrostomum lignano*. Dev Biol.

[33] Pfister D (2007). The exceptional stem cell system of *Macrostomum lignano*: Screening for gene expression and studying cell proliferation by hydroxyurea treatment and irradiation. Front Zool.

[34] Egger B (2009). The caudal regeneration blastema is an accumulation of rapidly proliferating stem cells in the flatworm *Macrostomum lignano*. BMC Dev Biol.

[35] Callow ME (2000). Use of self-assembled monolayers of different wettabilities to study surface selection and primary adhesion processes of green algal (Enteromorpha) zoospores. Appl Environ Microbiol.

[36] Ederth T (2008). Anomalous settlement behavior of *Ulva linza* zoospores on cationic oligopeptide surfaces. Biofouling.

[37] Ederth T (2011). Resistance of galactoside-terminated alkanethiol self-assembled monolayers to marine fouling organisms. ACS Appl Mater Interfaces.

[38] Rosenhahn A, Schilp S, Kreuzer HJ, Grunze M (2010). The role of “inert” surface chemistry in marine biofouling prevention. Phys Chem Chem Phys.

[39] Schilp S (2009). Physicochemical properties of (ethylene glycol)-containing self-assembled monolayers relevant for protein and algal cell resistance. Langmuir.

[40] Hwang DS (2010). Protein- and metal-dependent interactions of a prominent protein in mussel adhesive plaques. J Biol Chem.

[41] Kamino K, Smith AM (2016). Barnacle underwater attachment. Biological Adhesives.

[42] Wang CS, Stewart RJ (2012). Localization of the bioadhesive precursors of the sandcastle worm, *Phragmatopoma californica* (Fewkes). J Exp Biol.

[43] Zhao H, Sun C, Stewart RJ, Waite JH (2005). Cement proteins of the tube-building polychaete *Phragmatopoma californica*. J Biol Chem.

[44] Rodrigues M (2016). Profiling of adhesive-related genes in the freshwater cnidarian *Hydra magnipapillata* by transcriptomics and proteomics. Biofouling.

[45] Kamino K, Nakano M, Kanai S (2012). Significance of the conformation of building blocks in curing of barnacle underwater adhesive. FEBS J.

[46] Ohkawa K, Nishida A, Yamamoto H, Waite JH (2004). A glycosylated byssal precursor protein from the green mussel *Perna viridis* with modified dopa side-chains. Biofouling.

[47] Smith AM, Quick TJ, St Peter RL (1999). Differences in the composition of adhesive and non-adhesive mucus from the limpet *Lottia limatula*. Biol Bull.

[48] Stanley MS, Callow ME, Callow JA (1999). Monoclonal antibodies to adhesive cell coat glycoproteins secreted by zoospores of the green alga *Enteromorpha*. Planta.

[49] Hennebert E, Wattiez R, Flammang P (2011). Characterisation of the carbohydrate fraction of the temporary adhesive secreted by the tube feet of the sea star *Asterias rubens*. Mar Biotechnol (NY).

[50] Hermans CO (1983). The duo-gland adhesive system. Oceanogr Mar Biol Ann Rev.

[51] Thomas LA, Hermans CO (1985). Adhesive interactions between the tube feet of a starfish, *Leptasterias hexactis*, and substrata. Biol Bull.

[52] Flammang P, Michel A, Cauwenberge AV, Alexandre H, Jangoux M (1998). A study of the temporary adhesion of the podia in the sea star *Asterias rubens* (Echinodermata, asteroidea) through their footprints. J Exp Biol.

[53] Kearn GC, Evans-Gowing R (1998). Attachment and detachment of the anterior adhesive pads of the monogenean (platyhelminth) parasite *Entobdella soleae* from the skin of the common sole (*Solea solea*). Int J Parasitol.

[54] Lengerer B, Ladurner P (2018). Properties of temporary adhesion systems of marine and freshwater organisms. J Exp Biol.

[55] Kind M, Wöll C (2009). Organic surfaces exposed by self-assembled organothiol monolayers: Preparation, characterization, and application. Prog Surf Sci.

[56] Prime KL, Whitesides GM (1993). Adsorption of proteins onto surfaces containing end-attached oligo(ethylene oxide): A model system using self-assembled monolayers. J Am Chem Soc.

[57] Herrwerth S, Eck W, Reinhardt S, Grunze M (2003). Factors that determine the protein resistance of oligoether self-assembled monolayers—Internal hydrophilicity, terminal hydrophilicity, and lateral packing density. J Am Chem Soc.

[58] Harder P, Grunze M, Dahint R, Whitesides GM, Laibinis PE (1998). Molecular conformation in oligo(ethylene glycol)-terminated self-assembled monolayers on gold and silver surfaces determines their ability to resist protein adsorption. J Phys Chem B.

[59] Wang RLC, Kreuzer HJ, Grunze M (2000). The interaction of oligo(ethylene oxide) with water: A quantum mechanical study. Phys Chem Chem Phys.

[60] Wang RLC, Kreuzer HJ, Grunze M (1997). Molecular conformation and solvation of oligo(ethylene glycol)-terminated self-assembled monolayers and their resistance to protein adsorption. J Phys Chem B.

[61] Finlay JA, Callow ME, Ista LK, Lopez GP, Callow JA (2002). The influence of surface wettability on the adhesion strength of settled spores of the green alga *Enteromorpha* and the diatom *Amphora*. Integr Comp Biol.

[62] Ista LK (2004). Effect of substratum surface chemistry and surface energy on attachment of marine bacteria and algal spores. Appl Environ Microbiol.

[63] Schilp S (2007). Settlement and adhesion of algal cells to hexa(ethylene glycol)-containing self-assembled monolayers with systematically changed wetting properties. Biointerphases.

[64] Tseng YC, Tabata Y, Hyon SH, Ikada Y (1990). In vitro toxicity test of 2-cyanoacrylate polymers by cell culture method. J Biomed Mater Res.

[65] Burke SA, Ritter-Jones M, Lee BP, Messersmith PB (2007). Thermal gelation and tissue adhesion of biomimetic hydrogels. Biomed Mater.

[66] Li J (2017). Tough adhesives for diverse wet surfaces. Science.

[67] Rieger RM (1988). Laboratory cultures of marine Macrostomida (Turbellaria). Fortschr Zool.

[68] Clark K, Karsch-Mizrachi I, Lipman DJ, Ostell J, Sayers EW (2016). GenBank. Nucleic Acids Res.

[69] Kozlowski LP (2016). IPC—Isoelectric Point Calculator. Biol Direct.

[70] Finn RD (2017). InterPro in 2017—Beyond protein family and domain annotations. Nucleic Acids Res.

[71] Marchler-Bauer A (2017). CDD/SPARCLE: Functional classification of proteins via subfamily domain architectures. Nucleic Acids Res.

[72] Deutsch EW (2017). The ProteomeXchange Consortium in 2017: Supporting the cultural change in proteomics public data deposition. Nucleic Acids Res.

[73] Perez-Riverol Y (2019). The PRIDE database and related tools and resources in 2019: Improving support for quantification data. Nucleic Acids Res.

[74] Salvenmoser W, Egger B, Achatz JG, Ladurner P, Hess MW (2010). Electron microscopy of flatworms: Standard and cryo-preparation methods. Methods Cell Biol.

[75] Thiery J (1967). Mise en evidence des polysaccharides sur coupes fines en microscopie electronique. J Microsc (Paris).

[76] Svedhem S (2001). Synthesis of a series of oligo(ethylene glycol)-terminated alkanethiol amides designed to address structure and stability of biosensing interfaces. J Org Chem.

[77] Valiokas R, Svedhem S, Svensson SCT, Liedberg B (1999). Self-assembled monolayers of oligo(ethylene glycol)-terminated and amide group containing alkanethiolates on gold. Langmuir.

[78] Prime KL, Whitesides GM (1991). Self-assembled organic monolayers: Model systems for studying adsorption of proteins at surfaces. Science.

