# Supplementary material for: A mechanism for temporary bioadhesion
Source: Proc Natl Acad Sci U S A. 2019 Feb 19;116(10):4297–306. doi: 10.1073/pnas.1814230116 (PMC6410801; doi:10.1073/pnas.1814230116)
Supplement: Supplementary File [file pnas.1814230116.sapp.pdf]

PNAS
www.pnas.org

Supplementary Information for

A Mechanism for Temporary Bioadhesion

Julia Wunderer, Birgit Lengerer, Robert Pjeta, Philip Bertemes, Leopold Kremser, Herbert Lindner, Thomas Ederth, Michael W. Hess, David Stock, Willi Salvenmoser, Peter Ladurner

Corresponding author:

Peter Ladurner

Email: peter.ladurner@uibk.ac.at

This PDF file includes:

Figs. S1 to S19

Tables S1 to S3

Captions for movies S1 to S9

Other supplementary materials for this manuscript include the following:

Caption for Datasets D1 - D3

www.pnas.org/cgi/doi/10.1073/pnas.1814230116

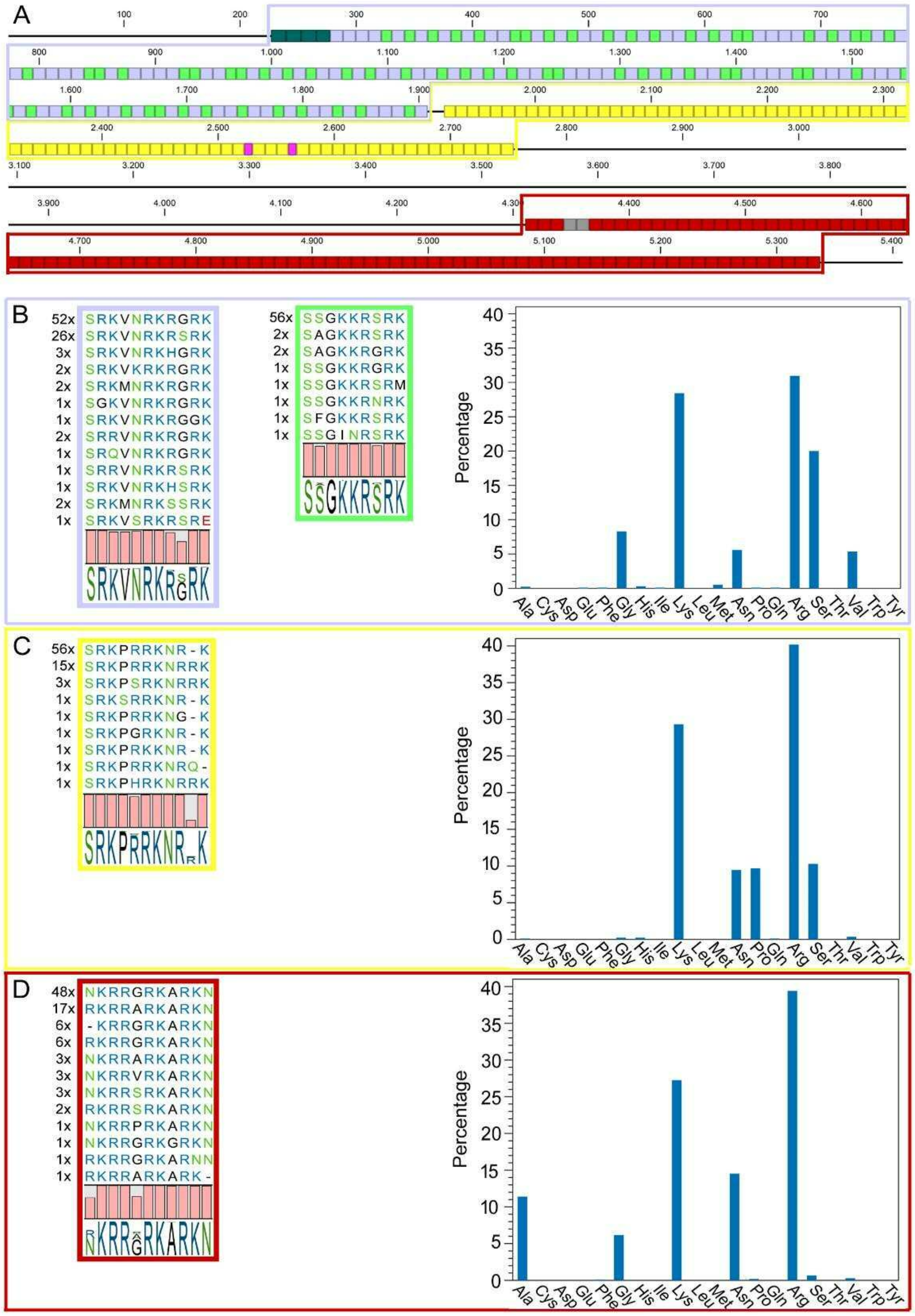

Fig. S1. Organization of Lysine-Arginine repeats in Mlig-ap1

(**A**) schematic illustration of Mlig-ap1 protein sequence. The three Lysine-Arginine rich repeat regions are highlighted in purple-green (KR region A), yellow (KR region B) and red (KR region C). (**B-D**) alignments of the different KR-rich repeats. Diagrams indicate the percentage of amino acids in the repeats. Amino acids are color-coded according to polarity colors: green neutral polar, black neutral nonpolar, red acidic polar, blue basic polar.

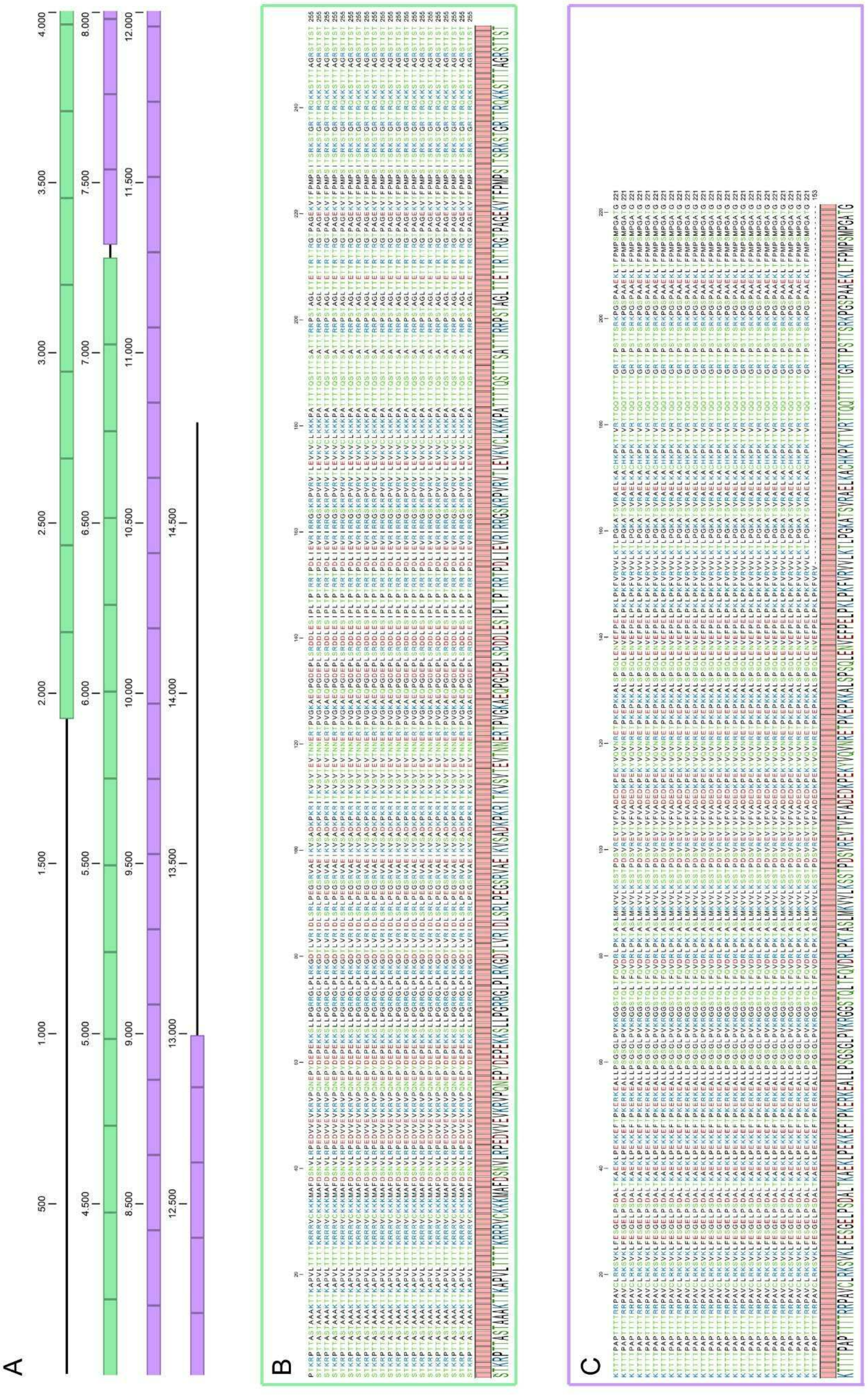
A
500
1.000
1.500
2.000
2.500
3.000
3.500
4.000
4.500
5.000
5.500
6.000
6.500
7.000
7.500
8.000
8.500
9.000
9.500
10.000
10.500
11.000
11.500
12.000
12.500
13.000
13.500
14.000
14.500
B
C

Fig. S2. Organization of Repeat A and Repeat B in Mlig-ap2

(**A**) schematic illustration of Mlig-ap2 protein sequence. Repeat region A is colored in green, repeat region B in purple. (**B**) alignment of repeat A, (**C**) alignment of repeat B. Amino acids are color-coded according to Polarity colors: green neutral polar, black neutral nonpolar, red acidic polar, blue basic polar.

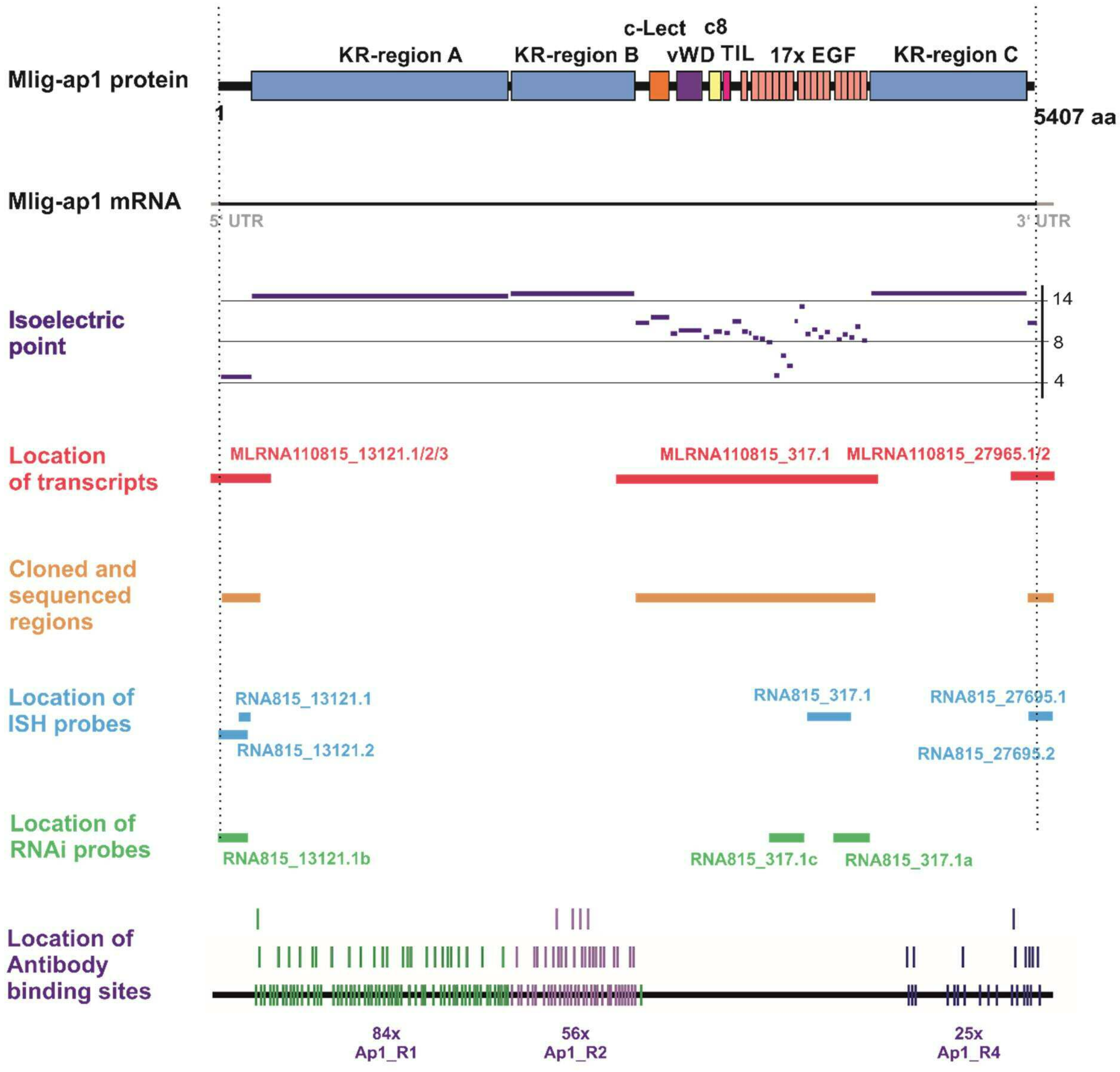

Fig. S3. Mlig-ap1 at a glance
Schematic illustration of Mlig-ap1 protein with the isoelectric point of protein regions and domains, location of according transcripts found in the transcriptome MLRNA110815, cloned regions, location of *in situ* hybridization probes and RNAi target sites as well as Antibody binding sites. *aa* amino acids, *C8* domain of 8 conserved Cysteines, *c-Lect* C-type lectin binding domain, *EGF* epidermal growth factor-like domain, *KR region* Lysine-Arginine rich repeat region, *TIL* Trypsin inhibitor-like domain, *vWD* von Willebrand domain.

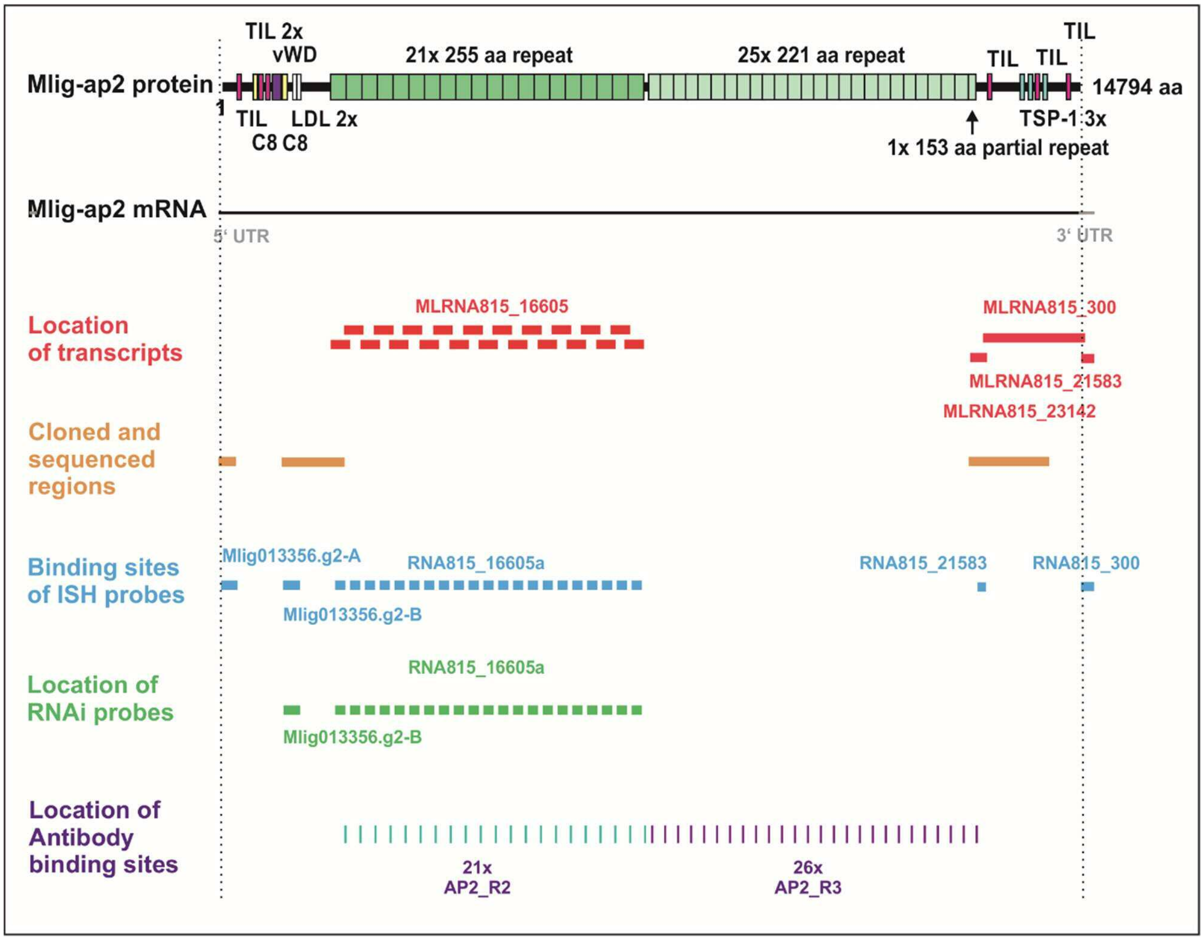

Fig. S4. Mlig-ap2 at a glance

Schematic illustration of Mlig-ap2 protein with the location of according transcripts found in the transcriptome MLRNA110815, cloned regions, location of *in situ* hybridization probes and RNAi target sites as well as antibody binding sites. *aa* amino acids, *C8* domain of 8 conserved Cysteines, *LDL* low-density lipoprotein receptor-like domain, *TSP-1* Thrombospondin-1-like domain, *TIL* Trypsin inhibitor-like domain, *vWD* von Willebrand domain.

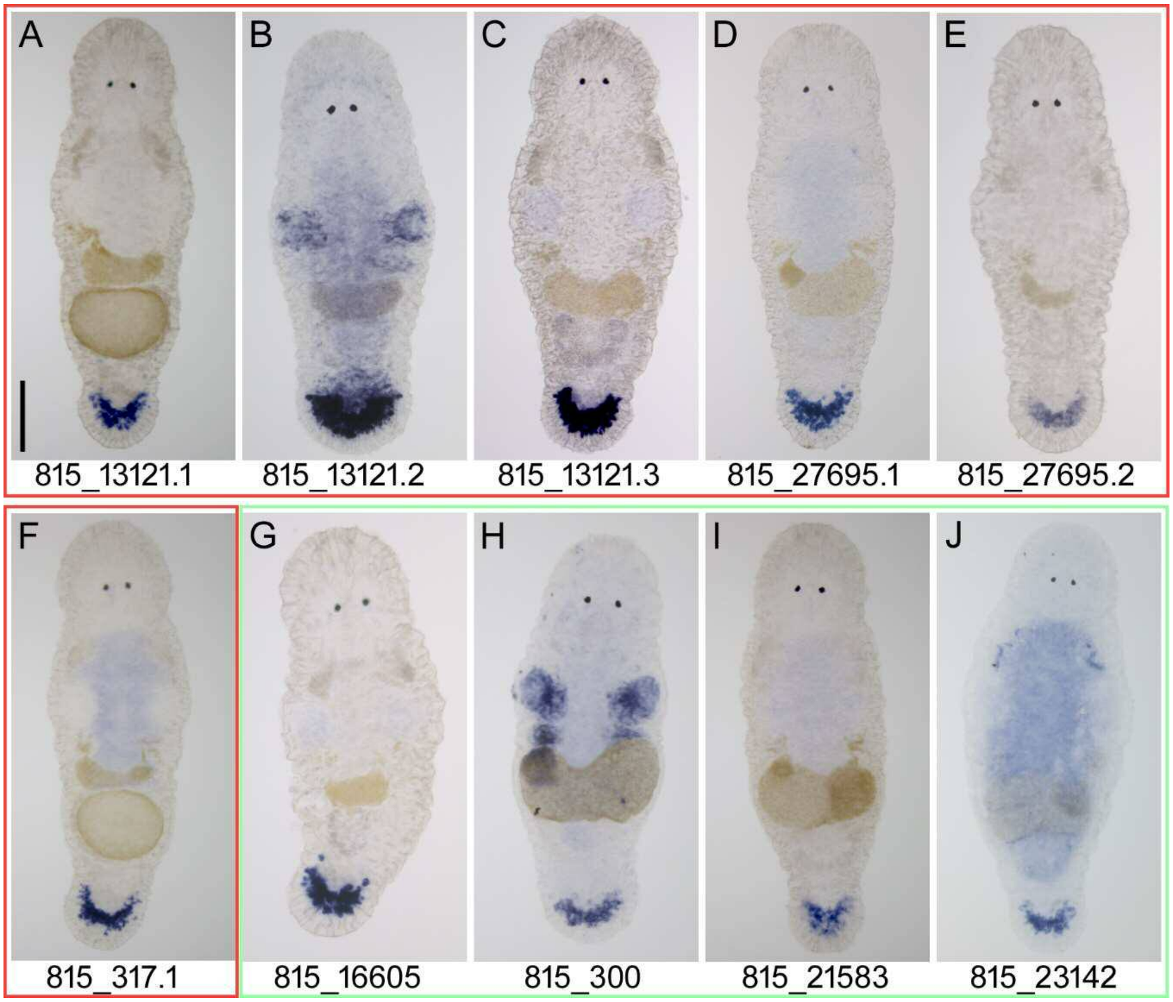

Fig. S5. Multiple *in situ* hybridization probes confirm the expression of both adhesive genes in adhesive organs

Independent transcripts of the MLRNA110815 transcriptome (1) are partial sequences of Mlig-ap1 (red frame) or Mlig-ap2 (green frame) and are expressed in the adhesive gland cells. This panel corroborates the expression of these transcripts in earlier *in situ* hybridization screens (2, 3). *In situ* hybridization with different probes belonging to Mlig-ap1 and Mlig-ap2. (**A-F**) *in situ* probes generated according to transcriptomic data that target Mlig-ap1 expression (red frame). (**G-J**) *in situ* probes made using transcripts all belonging to Mlig-ap2 (green frame). Scale bar: 100 µm.

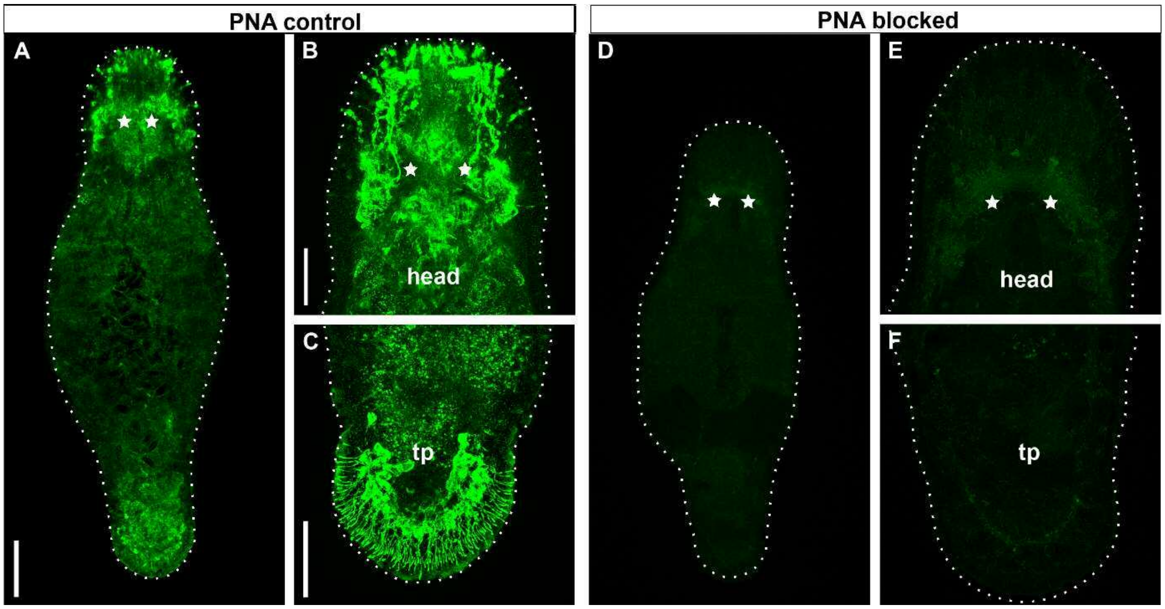

Fig. S6. Specificity of PNA labelling in *Macrostomum lignano*
Confocal projection (**A-C**) control PNA labelling with strong signal in frontal glands in the head region (**B**) and adhesive glands in the tail plate (**C**). (**D-E**) Labelling with PNA pre-incubated with 0.4 M D-galactose. Note that no fluorescent signal can be detected in the frontal glands (**E**) or the adhesive glands. Dotted white lines indicate the outline of the animals, white stars indicate the region of the eyes. *tp* tail plate. Scale bars: 100 µm (**A**), 50µm (**B, C**).

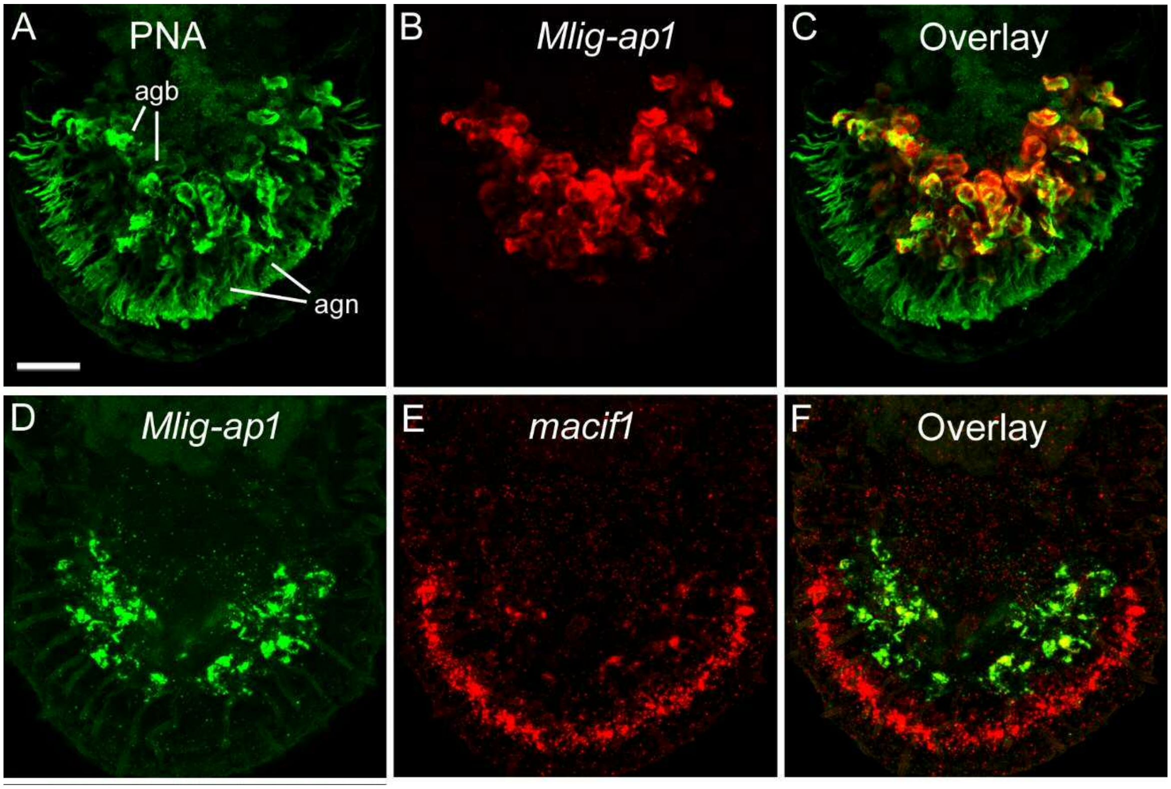

Fig. S7. Coexpression of Mlig-ap1 and Mlig-ap2

Confocal projection: (**A-C**) PNA labelling of Mlig-ap2 (green) combined with fluorescence in situ hybridization for *Mlig-ap1* (red). Note that the expression of *Mlig-ap1* RNA is restricted to adhesive gland cell bodies while PNA labels adhesive gland vesicles in the cell bodies and the adhesive gland necks. (**D-F**) double fluorescence *in situ* hybridization for *Mlig-ap1* (green, in adhesive gland cell bodies) and *macif1* (red, in anchor cells) (4). *agb* adhesive gland body, *agn* adhesive gland neck. Scale bar: 20 µm

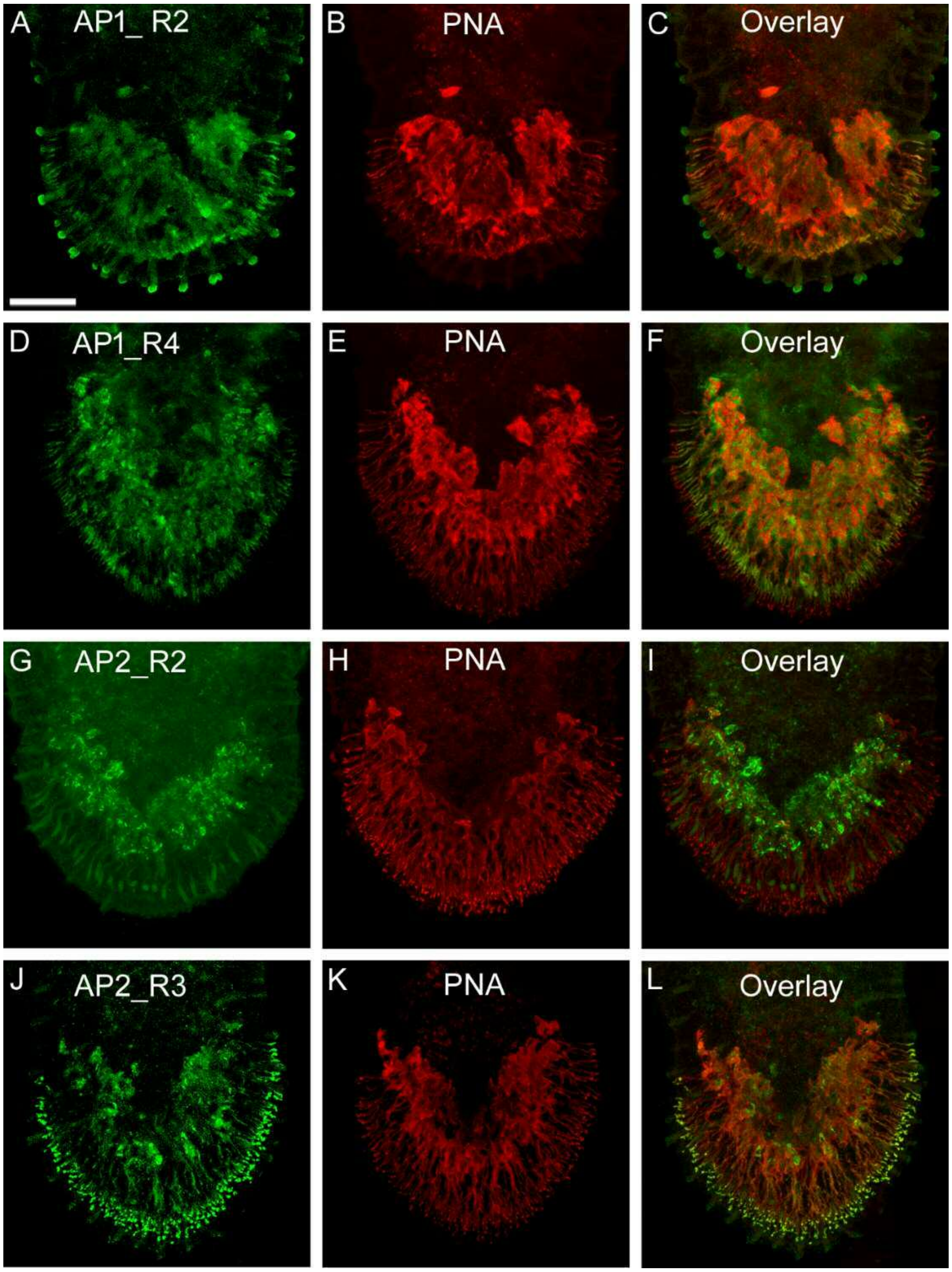

Fig. S8. Staining of Mlig-ap1 and Mlig-ap2 with Antibodies
Confocal projections of double labelling with antibodies against Mlig-ap1 or Mlig-ap2 combined with PNA staining. (**A-C**) antibody binding to Mlig-ap1 KR region B (green) and PNA (red). (**D-F**) Antibody raised against Mlig-ap1 KR region C and PNA. (**G-I**) antibody against Mlig-ap2 repeat 1 and PNA. (**J-L**) Antibody binding to Mlig-ap2 repeat 2 and PNA. For location of antibody binding sites of Mlig-ap1 and Mlig-ap2 see Figs. S3 and S4, respectively. Scale bar: 40 µm.

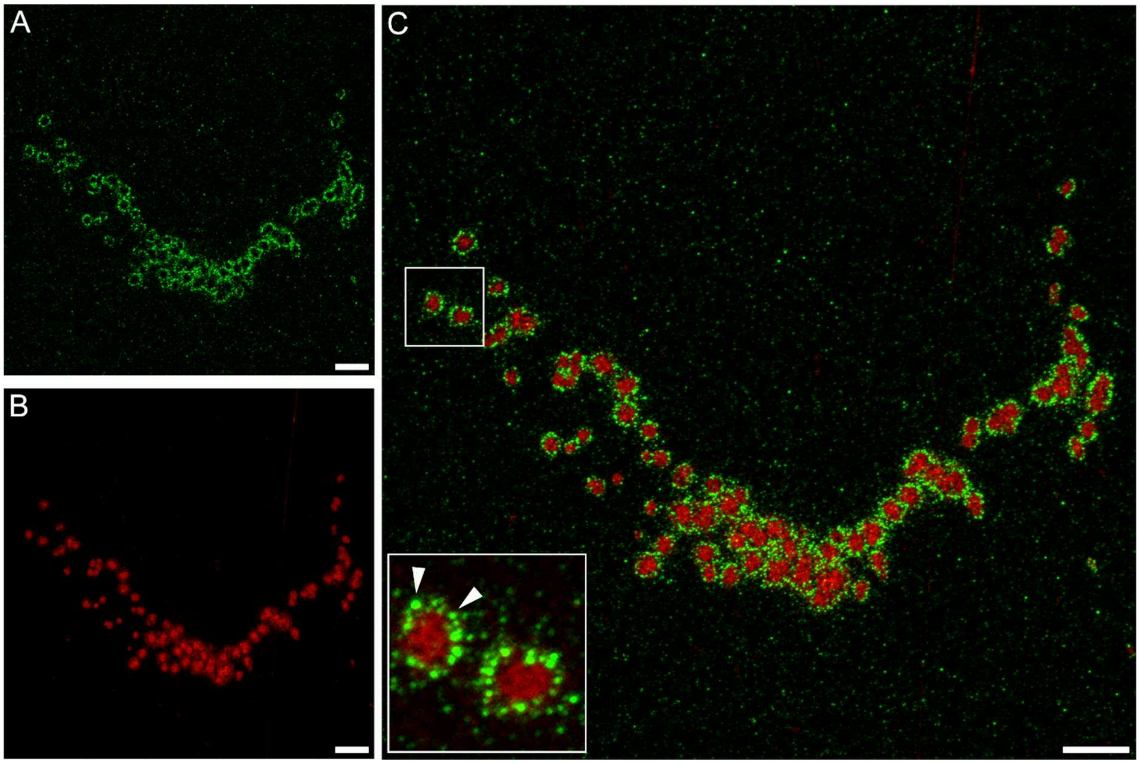

Fig. S9. Footprint labelled with Mlig-ap1 and Mlig-ap1
Confocal projection of double-labelled footprint with antibody against Mlig-ap1 KR region A (**A**) and PNA (**B**). White rectangle in the overlay (**C**) indicates the area of enlargement (inset). Note the dot-like labelling of Mlig-ap1 surrounding the PNA-positive Mlig-ap2 (white arrowheads). Scale bar: 10 µm.

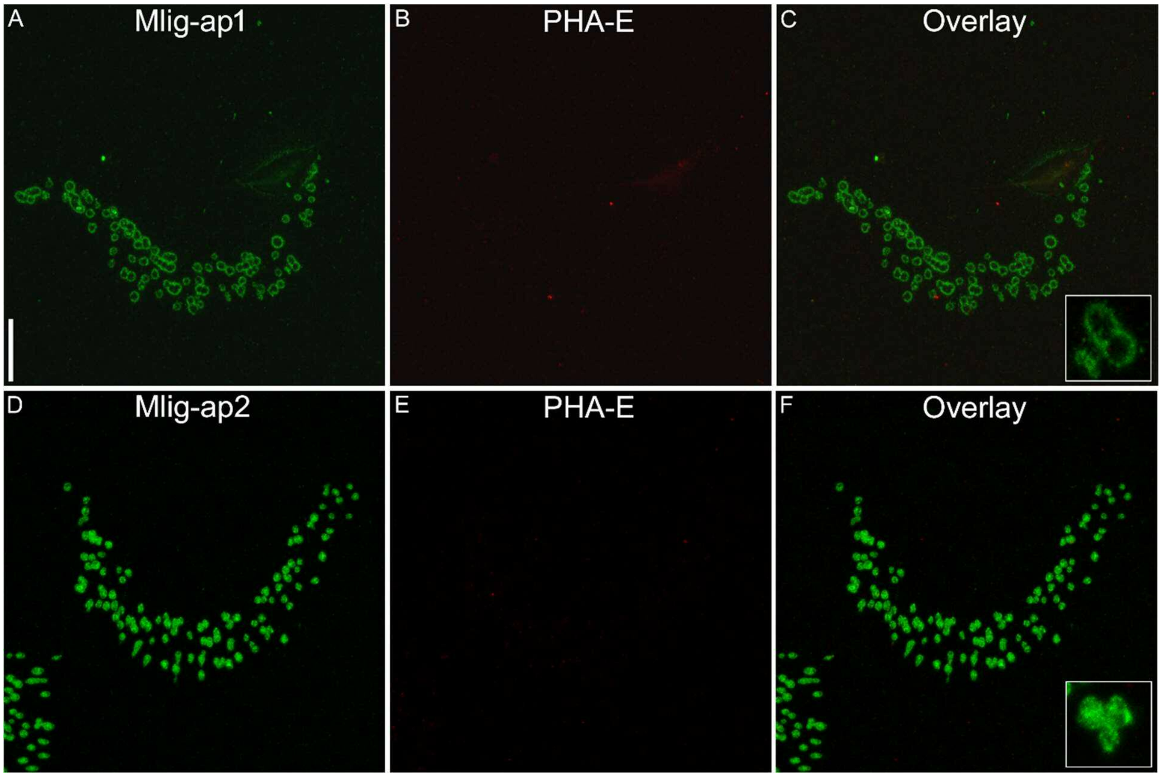

Fig. S10. Labelling of footprints after detachment

Confocal projection (**A-C**), footprints double-labelled with Antibody against Mlig-ap1 and PHA-E (4). (**D-F**) footprints double-labelled with Antibody against Mlig-ap2 and PHA-E. The lack of PHA-E staining indicated that no glycocalyx of the microvilli remained on the footprint. Scale bars: 20 µm.

MSASGVQKLGVLLLAVFCIGVTVASSEETVWGEWTEWSTSSGDAKSGDDVQSVVSTSWTEWVVDTSGGDSSSSSSSGSSRKRSGGGS
SEGSSSSGEWTTWTVVEDSSSSGQSSSGSRRRSDSTSDSQSGTSGSTNSGEWTTWTVVEDSSGSGHSSSGSSSRRSGSSRSRDDDAD
ELITEIIDETTIIDEDGYDITTGERNDRSSSARQQRGHSSSKSSGRKRSRMPSRKSSGRRRSRMSSRKSSGRRRSRMSSRKSSGKRN
SRMSSSKSSGRRRGRKSRKVNRKRGRKSRKVNRKRGRKSRKVNRKRGRKSRKVNRKRSRKSSGKKRSRKSRKVNRKRSRKSSGKKRG
RKSRKMNRKRGRKSSGKKRSRKSRKVNRKHSRKSSGKKRSRKSRKVNRKRGRKSSGKKRSRKSRKVNRKRGRKSRKVNRKRSRKSSG
KKRSRKSSGKKRSRKSRKVNRKRGRKSSGKKRSRKSRKVNRKRSRKSSGKKRSRKSRKVNRKRGRKSRKVNRKRGRKSAGKKRSRKS
RKVNRKRGRKSSGKKRSRKSRKVNRKRSRKSSGKKRSRKSRKVNRKRGRKSRKVNRKRGRKSSGKKRSRKSRKVSRKRSRESSGKKR
SRKSRKVNRKRSRKSSGKKRSRKSSGKKRSRKSRKVNRKRGRKSRKVNRKRGRKSRKVNRKRGRKSRKVNRKRSRKSSGKKRSRKSR
KVNRKRGRKSAGKKRGRKSRKMNRKSSRKSSGKKRSRKSSGKKRSRKSRRVNRKRGRKSSGKKRSRKSRKVNRKRGRKSRKVNRKRG
RKSSGKKRSRMSRKVNRKRGRKSRKVNRKRGRKSRKVKRKRGRKSRKVNRKRSRKSSGKKRSRKSSGKKRSRKSRKVNRKRGRKSSG
KKRSRKSRKVNRKRGRKSRKVNRKRGRKSRKVKRKRGRKSRKVNRKRSRKSSGKKRSRKSSGKKRSRKSRKVNRKRGRKSRKVNRKR
SRKSSGKKRSRKSSGKKRSRKSRKVNRKRSRKSSGKKRSRKSRKVNRKRSRKSSGINRSRKSRKVNRKRSRKSSGKKRSRKSRKVNR
KRGRKSRKVNRKRGRKSSGKKRSRKSRKVNRKRSRKSSGKKRSRKSRKVNRKRGRKSRKVNRKRGRKSSGKKRSRKSRKVNRKRGRK
SRKVNRKRGRKSSGKKRSRKSRKVNRKRGRKSSGKKRSRKSRKVNRKRGRKSSGKKRSRKSRKVNRKRSRKSSGKKRSRKSRKVNRK
RGRKSRKVNRKRGRKSSGKKRSRKSSGKKRSRKSRKVNRKRGRKSRKVNRKRGRKSRKVNRKRGRKSRRVNRKRSRKSSGKKRSRKS
RKVNRKRGRKSAGKKRGRKSRKMNRKSSRKSSGKKRSRKSRKVNRKRSRKSSGKKRSRKSRKVNRKHGRKSRKVNRKRSRKSSGKKR
SRKSSGKKRNRKSRRVNRKRGRKSRKVNRKRGRKSRKVNRKRGRKSRKMNRKRGRKSSGKKRSRKSSGKKRSRKSRKVNRKRGRKSR
KVNRKRGRKSRKVNRKRGRKSAGKKRSRKSRKVNRKRGRKSRKVNRKRGRKSRKVNRKRSRKSSGKKRSRKSRKVNRKRSRKSSGKK
RSRKSRKVNRKRGRKSRKVNRKRSRKSSGKKRSRKSRKVNRKRSRKSSGKKRSRKSRKVNRKHGRKSRKVNRKRGRKSSGKKRSRKS
RKVNRKHGRKSSGKKRSRKSSGKKRSRKSRKVNRKRGGKSFGKKRSRKSRKVNRKRGRKSSGKKRSRKSRKVNRKRGRKSRKVNRKR
SRKSSGKKRSRKSGKVNRKRGRKSRKVNRKRGRKSSGKKRSRKSRKVNRKRSRKSSGKKRSRKSRQVNRKRGRKSRKVNRKRSRKSS
GKKRSRKSRKVNRKRSRKSSGKKRSRKSRKVNRKRGRKSRKVNRKRGRKSRKVNRKRGRKSSGKKRSRKSRKVNRKRSRKSSGKKRS
HKPRKVKSRKPRRKNRKSRKPRRKNRKSRKSRRKNRKSRKPRRKNRKSRKPRRKNRKSRKPRRKNRRKSRKPRRKNRKSRKPRRKNG
KSRKPGRKNRKSRKPRRKNRRKSRKPRRKNRKSRKPRRKNRRKSRKPRKKNRKSRKPRRKNRKSRKPRRKNRKSRKPRRKNRKSRKP
RRKNRKSRKPRRKNRRKSRKPRRKNRQSRKPRRKNRRKSRKPRRKNRKSRKPRRKNRKSRKPSRKNRRKSRKPRRKNRKSRKPRRKN
RRKSRKPRRKNRKSRKPRRKNRKSRKPRRKNRKSRKPRRKNRKSRKPRRKNRKSRKPRRKNRKSRKPRRKNRKSRKPRRKNRRKSRK
PRRKNRKSRKPRRKNRKSRKPRRKNRKSRKPRRKNRRKSRKPRRKNRKSRKPRRKNRKSRKPRRKNRKSRKPRRKNRKSRKPSRKNR
RKSRKPRRKNRKSRKPRRKNRKSRKPRRKNRKSRKPRRKNRKSRKPRRKNRKSRKPRRKNRKSRKPRRKNRKSRKPRRKNRKSRKPR
RKNRKSRKPRRKNRKSRKPRRKNRKSRKPRRKNRKSRKPRRKNRKSRKPRRKNRRKSRKPRRKNRKSRKPRRKNRKSRKPRRKNRKS
RKPRRKSRKPRRKNRRKSRKPRRKNRKSRKPRRKSRKPRRKNRKSRKPRRKNRRKSRKPRRKNRKSRKPRRKNRKSRKPRRKNRKSR
KPRRKNRKSRKPRRKNRRKSRKPRRKNRKSRKPRRKNRRKSRKPRRKNRKSRKPHRKNRRKSRKPRRKNRKSRKPRRKNRKSRKPRR
KNRKSRKPRRKNRKSRKPRRKNRKSRKPSRKNRRKSRKPRRKNRRKSRKPRRKNRRKSRKVAVVSRNRRPRCRDRSSKCSDARKSGL
CKKIPLAMGKKCARTCGFCSKVARRGVPTCRDTAGSAVCQKWKARRYCQLYPKRMTMQCGLTCRLCSNNAKECPRGWRLAGSYCLRY
YSSPATSWRGAHRACQLATGELASVTSGKENRQLRAYLREVGAGRSRRSGGATWFGLIHPKSSRRPVFSDGSRLTSYARRRASKAAR
KTRGKCAYFVSGKWRFQSCKRKAGYVCRKRPGQQCSKACHKHGACSYVGRGRFQCKCKPGYFGDGVKSCRKPCTCVASGDPHYRTYD
GQWIHFMGKCTYTLTQLDSPNGASSPCQFDVRVRNHVVSREGLRRRKHRSTSLKSVSWTKFVEARVGKHTVQLGQDRRVLIDGERRS
LPVRNLGGGYNVFLDGPYVSLYSAQCGVKVSFNGRATAVIMVKPAYAGRMSGICGDCNGKRDDYTTRTGEDVSQRPNKFALVGNSYR
VPDEKRDKKLKARGDTCSDENDYFPECSASQLKRIRGSRLCGAIKDRAGPFAKCISRSKVDFSGYLSSCSYDVCVQLKQPKAVKRTI
CRVLAAIADECASAGYRARWRSYYGCPMKCPANSRYSFNTPSCQKTCRVNAVPRTQCPLTNQEGCVCRRGYVLSGRNCVRSGDCGCI
DEKGRYRKAGAAWVSDDCTKAYKCIGRRRIKSSRLRRCSKNAACRLRKGRRGCFCKRGYAGKAYGSRCRDIDECAAKKSPCHRNAKC
RNTPGSFVCRCRSGYSGDGKRSCKPVAEKKSACEKNPKVCGSHGVCKESDSNVRGYKCKCKPGYAPTGDGGRCEDVNECKTGKHRCH
RDARCINTPGSYGCVCNKNYEGDGRLVCKAKSACSSGQARCHRDAVCVDLPGGEYACRCKEGYRGDGRRLCEDINECSSGCKTCSVN
ADCINTPGSFRCVCRQGYAGDGIVCENVNECATGEARCSANADCIDTPGAYNCTCRRGFRGDGRTCRDVDECADGSDRCHKLADCIN
KVGSYACRCRDGYTGNGRQCRDVNECRLGTHTCSRKRARCVNRTGGYKCVCRAPFVGDGRQCRRRRNPKKPKTRRRRRKPKRVVKRR
SVKKPRKKPVVRCASGKARCHKNADCKDTKTGYTCRCRRGYYGDGYTCKDVNECKLGKHKCSRGKANCINKRGSYSCRCKPGYRGNG
FVCTDIDECRTGRDRCDPNASCRNKAGGYTCRCRRGFRGDGYNCADVDECRLGTNRCSRKADCRNTPGSYTCRCRKGYRGNGKVCRD
INECAEGKDRCHRFAQCINTPGSYRCRCKPGFYGNGRTCINRRQCADGTHKCSRYARCIQRNDGYDCKCNPGFRGNGKSCRDIDECK
AGTYRCHKDARCHNTLGSYTCRCNKGYRGDGYNTCKRIEVNECRDGTAKCSKNAVCIDRKSGYTCRCKAGYMGDGFTCTDVDECANK
KFPCPRGADCKNTPGSFSCRCKSGYRMMSGQCVDVNECREGKHRCSRNARCINKRGSYRCKCKKGFRGNGFVCKDIDECCGGKDRCD
PNAICINKIGSYKCKCLKGYVAKNGSCIPKKVPTKYIAVTKPKAKKFNKRRGRKARKNNKRRPRKARKNRKRRARKARKNRRRRARK
ARKNRRRRASKARKNRKRRSRKARKNRKRRARKARKNNKRRGRKARKNRKRRARKARKNKRRGRKARKNRKRRGRKARNNNKRRGRK
ARKNRKRRARKARKNKRRSRKARKNNKRRGRKARKNNKRRGRKARKNNKRRARKARKNNKRRGRKARKNNKRRGRKARKNNKRRGRK
ARKNNKRRARKARKNNKRRVRKARKNNKRRGRKARKNRKRRARKARKNKRRGRKARKNNKRRGRKARKNRKRRARKARKNKRRGRKA
RKNNKRRGRKARKNRKRRARKARKNNKRRGRKARKNRKRRARKARKNNKRRGRKARKNRKRRGRKARKNNKRRGRKARKNRKRRGRK
ARKNNKRRGRKARKNRKRRARKARKNNKRRGRKARKNNKRRSRKARKNRKRRGRKARKNRKRRSRKARKNNKRRGRKARKNNKRRSR
KARKNNKRRGRKARKNNKRRGRKARKNRKRRARKARKNNKRRGRKARKNNKRRGRKARKNNKRRGRKARKNRKRRARKARKNNKRRV
RKARKNNKRRGRKARKNRKRRGRKARKNNKRRGRKARKNRKRRARKARKNNKRRVRKARKNNKRRGRKARKNNKRRGRKARKNRKRR
ARKARKNNKRRGRKARKNRKRRARKARKNNKRRGRKARKNNKRRGRKARKNRKRRGRKARKNKRRGRKARKNRKRRGRKARKNNKRR
GRKARKNNKRRGRKARKNRKRRARKARKNRKRRARKARKNNKRRGRKARKNKRRGRKARKNNKRRARKARKNNKRRGRKARKNKRRG
RKARKNNKRRGRKARKNNKRRGRKARKNNKRRGRKARKNNKRRGRKARKNRKRRARKARKNNKRRGRKGRKNNKRR
GRKARKNNKRRGRKARKNNKRRGRKARKNNKRRGRKARKNNKRRGRKARKNNKRRGRKARKNRKRRARKARKNNKR
RGRKARKNNKRRGRKARKNRKRRGARKPRSGRKRARKARKSGLTKEKRSRKAKQAPRRQSSSQNSFGSSSGFDLGG
ATQFVSEANGFSE*

Fig. S11. Peptides found in mass spectrometry for Mlig-ap1

Mlig-ap1 protein sequence. Peptides found in mass spectrometry are indicated in grey. To highlight the K-R content, all lysines (K) (except the ones included in the mass spec peptides) are marked in green; all arginines (R) (except the ones included in the mass spec peptides) are labelled yellow.

Mlig-ap2:

MKLLTAVVAAQLLLACLTCGAAGAPEDSEPQWRYCLQTGLAHLLDFRGRWSSEFRPGGEAALASFTSEVLNWRVEVGTVGSGDC
GEDGAAEDACRKEIVIHFRGVPRCPTDTRVSIRGRSVRVVQLDTDGRNSSRTVAELRLGGDSEGDDDDDRLPASVPLLLRSFDS
GRQHSLHLSGTGLHLRWSDDGFLALLLESSSAAAAASSTGRTGGWCAGERPPPAPREGRTDAKRRRVRPANRGPCGRLDRPDGR
LARSCLRDSFVEVAGSAAAYATARRFLLSSRGRDCNSGGRSGAACRDGRAVLSLCHRRCPDRCAALRRLPPLMCALAPLCACAP
PRLLDSRGRCVEEAECPCRHGNRIYHRGERFTKDCQTCSCLGNNNWRCASSDCGRVCLATPGQVVTFDGQKISFVDNRNDYYLI
EPDDSKIPLAVKFSRSSPARLEVSWQGQQVLVYHRPATDSESGDFFIQLGDRSQKLAIGEHLRVGLGFFLHRVTSHYIRLRVAD
LLRVDFNGGTVELAASRRLRGARRLRGLCGRYDGSSENDLWPRRGRAAADSLSVARSYLVKAISPAGGPKRTTISREAVRTCYD
LFQHSGQFQRCARTENLQPFLRNCEAAGDRDSVCYIAFNAMRLCARSGYKIHWRLLPQLRNCALLACPRGGSSFRLCANTCQAT
CASLARPDRCPSGCYFGCQCPRGRYLMADGRCVTKSQCTCFDSATGRQRQPGEKFSRRNENCKCQDGRAVCQAAQSASANGRVA
CPKNQVWRRAQPGECWPRRCEDPVKDGSRCQSPDSAAEERCRCPAGLFATDSGLCVNRSDCPCRYGGRELLTGSVLTAQCPGRV
CSSGRWLNRAASESSSDSGCRGRCRAFGDGYYVSFDGRAFRYYAGSRETRLLQLPQLRVATRSVACGSSRPSVCARLVKIRLAG
GDEIRVLQGRIIAGLRHVDGEAVVLTATAALGGLHFLRIGVSIVWDRGTRLTINAARPRSGQPIGGLCGSFNGDATDDYRTPGG
LLALSPDEFGRSWTLESDYAQPIPESEYDQGCLKSRENFDWSFARCGLLRDQRGPFGRCLSVLNEDRLEFLHDACLREACRCRR
TVKDAANCDGLCNLMAAAADECAGLNVTVDWRSAAFCPYNCPEGTAYTSCGQLQSPFVFANGPDVQCLEDCRCRMGGQWTQAAG
SCTEASHVCRYRAAVYQTGDSAKLGCRQCTCREGKWIRCDNKDCYRNTTIELNNLSTVAPCTGHSFACRNSDACLHRSKVCDGV
PDCSDGSDEMSCEGCPAFKLKCRRSNLCIAKSEVCDGKHQCRLGNSTDDSDEENCATGATTSAPQKKPIEGTDSCYRPLLQSGL
IKESQVRITENFAPTHGYRRAELLDSRKRPYLLSKGRTVFELRLSPAGSAVRVSSVLLQIQSNHRRGLKAIRLYANSNKTWQLL
EAKESNIACTRCLIKLNKLPFAAEKLKFKIQSRSSMKIQLEINGCPGQNLESGRCRRLPATACIVKVYKSITYNREYSGEEKSR
LVLPRTEYSLLNQGSSEIVIGVPEMVRICAVIIKSKLPSDGKPTANVVSFVYDKKLSELTLVSKLPISTNTTTITISKPTNEYK
AVLYSKDPIRISIEIQVKKQPVTKNEPKQQKIACKGSLPDSEVSGKVRVLRMTALDSRQEYTQSDRRQLLRKGAALSSGRTDVL
VQFSLKQTDVIEALQVLTTTPGADAKVTARELQLCTRDRCSKVKMSTPSVQQTNMVIRARARFLKIILDSPTSRYAKNAKLKFD
FCSRNKPHVCKERYLERCNVPLMKSITFGKRYDAYTVKALLQGRPVRLLRGYTKITLINLNLAESVILKKTGGRRPLKLIISLT
TTNERKRKTTITKFDTVYSAKLPTGYTKMIITIKSKEPSKLALFKKECQKPNKIPNREPKTAAHTTTIAPATTTLEPTKRPTTA
STAAAKTTKAPVLTTTTKRRRVCKKKMAFDSNVLRPEDVVEVKRVPQNEPYDEPEKKSLLPGRRGLPLRKGDTLVRIDLSRLPE
GSRVAEIKVSADKPKRITKVSVTEVTNNERTPVGKAEQPGDEPLSRDDLESIPLTPTRRTPDLIEVRIRRGSKRPVRVTLEVKV
CLKKKPATTTTQSTTTSATTTRRPSTAGLTTETTRTTRGTPAGEKVTFPMPSITSRKSTGRTTRQKKSTTTAGRSTTSTSTKRP
TTASTAAAKTTKAPVLTTTTKRRRVCKKKMAFDSNVLRPEDVVEVKRVPQNEPYDEPEKKSLLPGRRGLPLRKGDTLVRIDLSR
LPEGSRVAEIKVSADKPKRITKVSVTEVTNNERTPVGKAEQPGDEPLSRDDLESIPLTPTRRTPDLIEVRIRRGSKRPVRVTLE
VKVCLKKKPATTTTQSTTTSATTTRRPSTAGLTTETTRTTRGTPAGEKVTFPMPSITSRKSTGRTTRQKKSTTTAGRSTTSTST
KRPTTASTAAAKTTKAPVLTTTTKRRRVCKKKMAFDSNVLRPEDVVEVKRVPQNEPYDEPEKKSLLPGRRGLPLRKGDTLVRID
LSRLPEGSRVAEIKVSADKPKRITKVSVTEVTNNERTPVGKAEQPGDEPLSRDDLESIPLTPTRRTPDLIEVRIRRGSKRPVRV
TLEVKVCLKKKPATTTTQSTTTSATTTRRPSTAGLTTETTRTTRGTPAGEKVTFPMPSITSRKSTGRTTRQKKSTTTAGRSTTS
TSTKRPTTASTAAAKTTKAPVLTTTTKRRRVCKKKMAFDSNVLRPEDVVEVKRVPQNEPYDEPEKKSLLPGRRGLPLRKGDTLV
RIDLSRLPEGSRVAEIKVSADKPKRITKVSVTEVTNNERTPVGKAEQPGDEPLSRDDLESIPLTPTRRTPDLIEVRIRRGSKRP
VRVTLEVKVCLKKKPATTTTQSTTTSATTTRRPSTAGLTTETTRTTRGTPAGEKVTFPMPSITSRKSTGRTTRQKKSTTTAGRS
TTSTSTKRPTTASTAAAKTTKAPVLTTTTKRRRVCKKKMAFDSNVLRPEDVVEVKRVPQNEPYDEPEKKSLLPGRRGLPLRKGD
TLVRIDLSRLPEGSRVAEIKVSADKPKRITKVSVTEVTNNERTPVGKAEQPGDEPLSRDDLESIPLTPTRRTPDLIEVRIRRGS
KRPVRVTLEVKVCLKKKPATTTTQSTTTSATTTRRPSTAGLTTETTRTTRGTPAGEKVTFPMPSITSRKSTGRTTRQKKSTTTA
GRSTTSTSTKRPTTASTAAAKTTKAPVLTTTTKRRRVCKKKMAFDSNVLRPEDVVEVKRVPQNEPYDEPEKKSLLPGRRGLPLR
KGDTLVRIDLSRLPEGSRVAEIKVSADKPKRITKVSVTEVTNNERTPVGKAEQPGDEPLSRDDLESIPLTPTRRTPDLIEVRIR
RGSKRPVRVTLEVKVCLKKKPATTTTQSTTTSATTTRRPSTAGLTTETTRTTRGTPAGEKVTFPMPSITSRKSTGRTTRQKKST
TTAGRSTTSTSTKRPTTASTAAAKTTKAPVLTTTTKRRRVCKKKMAFDSNVLRPEDVVEVKRVPQNEPYDEPEKKSLLPGRRGL
PLRKGDTLVRIDLSRLPEGSRVAEIKVSADKPKRITKVSVTEVTNNERTPVGKAEQPGDEPLSRDDLESIPLTPTRRTPDLIEV
RIRRGSKRPVRVTLEVKVCLKKKPATTTTQSTTTSATTTRRPSTAGLTTETTRTTRGTPAGEKVTFPMPSITSRKSTGRTTRQK
KSTTTAGRSTTSTSTKRPTTASTAAAKTTKAPVLTTTTKRRRVCKKKMAFDSNVLRPEDVVEVKRVPQNEPYDEPEKKSLLPGR
RGLPLRKGDTLVRIDLSRLPEGSRVAEIKVSADKPKRITKVSVTEVTNNERTPVGKAEQPGDEPLSRDDLESIPLTPTRRTPDL
IEVRIRRGSKRPVRVTLEVKVCLKKKPATTTTQSTTTSATTTRRPSTAGLTTETTRTTRGTPAGEKVTFPMPSITSRKSTGRTT
RQKKSTTTAGRSTTSTSTKRPTTASTAAAKTTKAPVLTTTTKRRRVCKKKMAFDSNVLRPEDVVEVKRVPQNEPYDEPEKKSLL
PGRRGLPLRKGDTLVRIDLSRLPEGSRVAEIKVSADKPKRITKVSVTEVTNNERTPVGKAEQPGDEPLSRDDLESIPLTPTRRT
PDLIEVRIRRGSKRPVRVTLEVKVCLKKKPATTTTQSTTTSATTTRRPSTAGLTTETTRTTRGTPAGEKVTFPMPSITSRKSTG
RTTRQKKSTTTAGRSTTSTSTKRPTTASTAAAKTTKAPVLTTTTKRRRVCKKKMAFDSNVLRPEDVVEVKRVPQNEPYDEPEKK
SLLPGRRGLPLRKGDTLVRIDLSRLPEGSRVAEIKVSADKPKRITKVSVTEVTNNERTPVGKAEQPGDEPLSRDDLESIPLTPT
RRTPDLIEVRIRRGSKRPVRVTLEVKVCLKKKPATTTTQSTTTSATTTRRPSTAGLTTETTRTTRGTHGGGGEKVTFPMPSITS
RKSTGRTTRQKKSTTTAGRSTTSTSTKRPTTASTAAAKTTKAPVLTTTTKRRRVCKKKMAFDSNVLRPEDVVEVKRVPQNEPYD
EPEKKSLLPGRRGLPLRKGDTLVRIDLSRLPEGSRVAEIKVSADKPKRITKVSVTEVTNNERTPVGKAEQPGDEPLSRDDLESI
PLTPTRRTPDLIEVRIRRGSKRPVRVTLEVKVCLKKKPATTTTQSTTTSATTTRRPSTAGLTTETTRTTRGTPAGEKVTFPMPS
ITSRKSTGRTTRQKKSTTTAGRSTTSTSTKRPTTASTAAAKTTKAPVLTTTTKRRRVCKKKMAFDSNVLRPEDVVEVKRVPQNE
PYDEPEKKSLLPGRRGLPLRKGDTLVRIDLSRLPEGSRVAEIKVSADKPKRITKVSVTEVTNNERTPVGKAEQPGDEPLSRDDL
ESIPLTPTRRTPDLIEVRIRRGSKRPVRVTLEVKVCLKKKPATTTTQSTTTSATTTRRPSTAGLTTETTRTTRGTPAGEKVTFP
MPSITSRKSTGRTTRQKKSTTTAGRSTTSTSTKRPTTASTAAAKTTKAPVLTTTTKRRRVCKKKMAFDSNVLRPEDVVEVKRVP
QNEPYDEPEKKSLLPGRRGLPLRKGDTLVRIDLSRLPEGSRVAEIKVSADKPKRITKVSVTEVTNNERTPVGKAEQPGDEPLSR
DDLESIPLTPTRRTPDLIEVRIRRGSKRPVRVTLEVKVCLKKKPATTTTQSTTTSATTTRRPSTAGLTTETTRTTRGTPAGEKV
TFPMPSITSRKSTGRTTRQKKSTTTAGRSTTSTSTKRPTTASTAAAKTTKAPVLTTTTKRRRVCKKKMAFDSNVLRPEDVVEVK
RVPQNEPYDEPEKKSLLPGRRGLPLRKGDTLVRIDLSRLPEGSRVAEIKVSADKPKRITKVSVTEVTNNERTPVGK

continued

AEQPGDEPLSRDDLESIPLTPTRRTPDLIEVRIRRGSKRPVRVTLEVKVCLKKKPATTTTQSTTTSATTTRRPSTAGLTTETTR
TTRGTPAGEKVTFPMPSITSRKSTGRTTRQKKSTTTAGRSTTSTSTKRPTTASTAAAKTTKAPVLTTTTKRRRVCKKKMAFDSN
VLRPEDVVEVKRVPQNEPYDEPEKKSLLPGRRGLPLRKGDTLVRIDLSRLPEGSRVAEIKVSADKPKRITKVSVTEVTNNERTP
VGKAEQPGDEPLSRDDLESIPLTPTRRTPDLIEVRIRRGSKRPVRVTLEVKVCLKKKPATTTTQSTTTSATTTRRPSTAGLTTE
TTRTTRGTPAGEKVTFPMPSITSRKSTGRTTRQKKSTTTAGRSTTSTSTKRPTTASTAAAKTTKAPVLTTTTKRRRVCKKKMAF
DSNVLRPEDVVEVKRVPQNEPYDEPEKKSLLPGRRGLPLRKGDTLVRIDLSRLPEGSRVAEIKVSADKPKRITKVSVTEVTNNE
RTPVGKAEQPGDEPLSRDDLESIPLTPTRRTPDLIEVRIRRGSKRPVRVTLEVKVCLKKKPATTTTQSTTTSATTTRRPSTAGL
TTETTRTTRGTPAGEKVTFPMPSITSRKSTGRTTRQKKSTTTAGRSTTSTSTKRPTTASTAAAKTTKAPVLTTTTKRRRVCKKK
MAFDSNVLRPEDVVEVKRVPQNEPYDEPEKKSLLPGRRGLPLRKGDTLVRIDLSRLPEGSRVAEIKVSADKPKRITKVSVTEVT
NNERTPVGKAEQPGDEPLSRDDLESIPLTPTRRTPDLIEVRIRRGSKRPVRVTLEVKVCLKKKPATTTTQSTTTSATTTRRPST
AGLTTETTRTTRGTPAGEKVTFPMPSITSRKSTGRTTRQKKSTTTAGRSTTSTSTKRPTTASTAAAKTTKAPVLTTTTKRRRVC
KKKMAFDSNVLRPEDVVEVKRVPQNEPYDEPEKKSLLPGRRGLPLRKGDTLVRIDLSRLPEGSRVAEIKVSADKPKRITKVSVT
EVTNNERTPVGKAEQPGDEPLSRDDLESIPLTPTRRTPDLIEVRIRRGSKRPVRVTLEVKVCLKKKPATTTTQSTTTSATTTRR
PSTAGLTTETTRTTRGTPAGEKVTSPMPSITSRKSTGRTTRQKKSTTTAGRSTTSTSTKRPTTASTAAAKTTKAPVLTTTTKRR
RVCKKKMAFDSNVLRPEDVVEVKRVPQNEPYDEPEKKSLLPGRRGLPLRKGDTLVRIDLSRLPEGSRVAEIKVSADKPKRITKV
SVTEVTNNERTPVGKAEQPGDEPLSRDDLESIPLTPTRRTPDLIEVRIRRGSKRPVRVTLEVKVCLKKKPATTTTQSTTTSATT
TRRPSTAGLTTETTRTTRGTPAGEKVTFPMPSITSRKSTGRTTRQKKSTTTAGRSTTSTSTKRPTTASTAAARTTNASLLTMKT
TKILPSIRVSSRRKTTTTPAPTTTTRRPAVCLRKSVKLFESGELPSDALTKAEKLPEKKEFTPKERKEALLPSGSGLPVKRGGS
TQLTFQVDRLPKTASLMKVVLKSSTPDSVREVTVFVADEDKPEKYVQVNRETPKEPKKALSPSQLENVEFPELPKLPKFVRVVL
KTLPGKATSVRAELKACHKPKTTVRTTQQTTTTTGRTTPSTTSRKPGSPAAEKLTFPMPSMPGATGKTTTTPAPTTTTRRPAVC
LRKSVKLFESGELPSDALTKAEKLPEKKEFTPKERKEALLPSGSGLPVKRGGSTQLTFQVDRLPKTASLMKVVLKSSTPDSVRE
VTVFVADEDKPEKYVQVNRETPKEPKKALSPSQLENVEFPELPKLPKFVRVVLKTLPGKATSVRAELKACHKPKTTVRTTQQTT
TTTGRTTPSTTSRKPGSPAAEKLTFPMPSMPGATGKTTTTPAPTTTTRRPAVCLRKSVKLFESGELPSDALTKAEKLPEKKEFT
PKERKEALLPSGSGLPVKRGGSTQLTFQVDRLPKTASLMKVVLKSSTPDSVREVTVFVADEDKPEKYVQVNRETPKEPKKALSP
SQLENVEFPELPKLPKFVRVVLKTLPGKATSVRAELKACHKPKTTVRTTQQTTTTTGRTTPSTTSRKPGSPAAEKLTFPMPSMP
GATGKTTTTPAPTTTTRRPAVCLRKSVKLFESGELPSDALTKAEKLPEKKEFTPKERKEALLPSGSGLPVKRGGSTQLTFQVDR
LPKTASLMKVVLKSSTPDSVREVTVFVADEDKPEKYVQVNRETPKEPKKALSPSQLENVEFPELPKLPKFVRVIVQILAEMSTN
VSLDLFSCQDDFYPCMNCTHNGTSYIEGSIVSLNGVKCDHHVCRKRSWEPLYDCSSWSSWSNCSSECDAGFKTRMKICRSSFRD
LIARQNLTSLEKCFNQVEREPCFNRRCSKTECNPAEWGPWSPCSGECSTLLSERKRQPGSAVLSEFCKKVQLPESRLCPKTEKC
LCKDPNAAWNNCSNQCPFTCGHLSEQSACVMTSACVPGCACKPGYYLQDGRCVPQSQCRCPGLTPTTLAISTTALTTPSTGAAA
ALFVSYSSPKARIRGTAAPDKISMLIPDLNLLIPALPTIVGGGASKPADGADLKSTSVSVGAKTNPPATSSSQAPRIRGTAAPD
RASMLIPMLPGKPAATPVKPATTATAPTPTTPVRIRGTAAPDGVNLLIPTLSLGSAANTTTMAPSTTVPTTTAAASVSTVASVN
GIPAAPSNAATPGAFAPGVTSGSSGSFTVKGFVSPAQGLGKANTMGAFRVTSLLNMKRPPPPPPPRPGGNHGRFQHSAFRLPPK
FHLPAPSGGRPAFRLPGQFKPPPAFRPPGGGSFGKFNPGKPVFRLPGAGSFNPPGKPGTKNSSPSVPHTPAAASPAQPVTPKSS
ASTPAPPPGLKPVPSGQQASVAPTKPTTKAPVPMSSQGTRIRGTAADDRVDLRIPTLPNLLPLPTSPRTKLFTKRSTPSTRSMP
SIGPHTSATAKRKLTSAAPVTSKPLRPSFPGIPPAGPHAPPGGQGSSPATPKKPTTVVTTTSMGTRATNSWVMIGGSNCTCTSG
KTTCVGGHEDSLAAWSTWTAWSNCSAHCGQGKQYRFRQCSGPACVGNGHANESRACTGVDCYCVFSRAKLQWLLTDDYYHAEMW
VERDGKPGLSSPLETVNDGDRLESGSVLQVGCLRCTCKDGGFACKLQRCASDCQWSAWQPWATCSATCGGGLQTRLRTIHKPAV
ADGAPCQASGFTDTQACNLDPCPGCSWSSWSQWSNCTGSATSDIGVRERRRQPIGSCKEADRDLQPCQLRPAQSKCKPGQLHSA
CHNVTGCEVACSAGANARTLCSQTDACLSGCLCPKGQVMGPNGQCVATDKCRCLPKSINCSNCFKPVCLNGQPTCLPDMNCQRL
YTEWSAWSACSAACGEGLSHRTRQLLQEGRRPAGCGKSDLIETRHCNARPCGYACKDRNGDLYTEGQAMPALDQCSMCFCRANR
TECVSKAKVGEPVPTPVWSAWSTWSSCVSAAANNRRQERRRRCVRICPDDPRKCGEFFDKEESVEWKACLPKTPLLKRDCIVSP
DWIEGTCYANCMVRSAVARGFRTLYRRILQLPANGGAPCPELRREDVPCTKTCPVDCSQTAWSAWGACVRASNSGCSGLQVRSR
GVLVPPANGGRACEDAKQLRSCLAPGCQTSACRAPMTKQPLQQSRCIVRCSDLASEDKCAPVQAANAAAVNATVCACPAGQYEQ
DGRCVAKQACRCRWNEELMGQRSPGTREEFAAGERLVMGCKNCTCQDGVFKCNRLSSCSDECHWSNWRPEGKCTAGCRLKPGLQ
KFVRSPRNSDYLPTKACQGPSVKYELCSSTRSCDSLCPSGQRYNEFGECDEQCYSAGSLGQPQRQRCPEQACVCARGLVKRGDS
CVPKSECYKCSSGNTTLPDGGYSVDLAACQIVTCDKGRTSAVPLSVDQMPACSLEDERIAQSAPKAKDPNRCCYRASASACSRE
EVSISKLDDSCLLTSPIRVPHCRGQCPQASPLIAPRRSDVVAEVAAADSSQVYLLKLPGAHCTGCLPDRVQYSPTTLTVKCKS
GESRQLRMPIVGACHCKQVPCPSTA

Fig. S12. Peptides found in mass spectrometry for Mlig-ap2

Peptides identified by Mass Spectrometry. Color code: Footprints Trypsin; Footprints LysC; Footprints V8

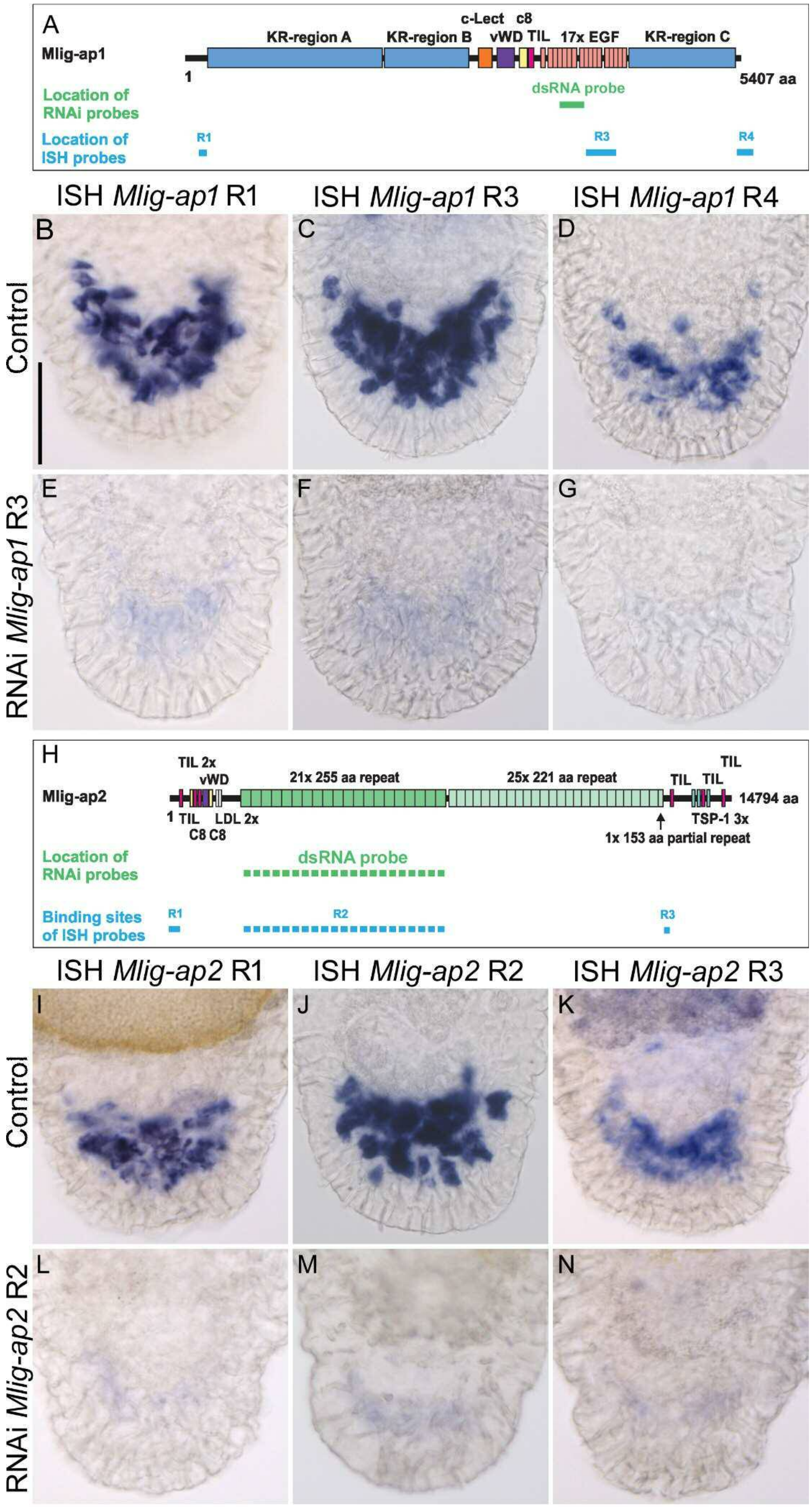

A
Mlig-ap1
KR-region A
KR-region B
c-Lect
vWD
c8
TIL
17x EGF
KR-region C
1
5407 aa
Location of RNAi probes
dsRNA probe
Location of ISH probes
R1
R3
R4
ISH Mlig-ap1 R1
ISH Mlig-ap1 R3
ISH Mlig-ap1 R4
B
C
D
Control
E
F
G
RNAi Mlig-ap1 R3
H
Mlig-ap2
TIL 2x
vWD
21x 255 aa repeat
25x 221 aa repeat
TIL
TIL
TIL
14794 aa
1
TIL
C8 C8
LDL 2x
TSP-1 3x
1x 153 aa partial repeat
Location of RNAi probes
dsRNA probe
Binding sites of ISH probes
R1
R2
R3
ISH Mlig-ap2 R1
ISH Mlig-ap2 R2
ISH Mlig-ap2 R3
I
J
K
Control
L
M
N
RNAi Mlig-ap2 R2

Fig. S13. RNAi knock-down of Mlig-ap1 and Mlig-ap2

(**A**) schematic illustration of Mlig-ap1 protein with location of RNAi probes and different *in situ* hybridization probes. (**B-G**) RNAi experiment for Mlig-ap1. *Mlig-ap1* expression in the tail region shown by three different *in situ* hybridization probes (R1, R2, R4) in control animals (**B-D**) and dsRNA treated animals (**E-G**). (**H**) schematic illustration of Mlig-ap2 protein with location of dsRNA probe and *in situ* hybridization probes (R1, R2, R3). (**I-N**) RNAi experiment for Mlig-ap2. *In situ* hybridization in the tail region against three different regions of Mlig-ap2 in control animals (**I-K**) and dsRNA treated animals (**L-N**). Scale bar: 50 µm.

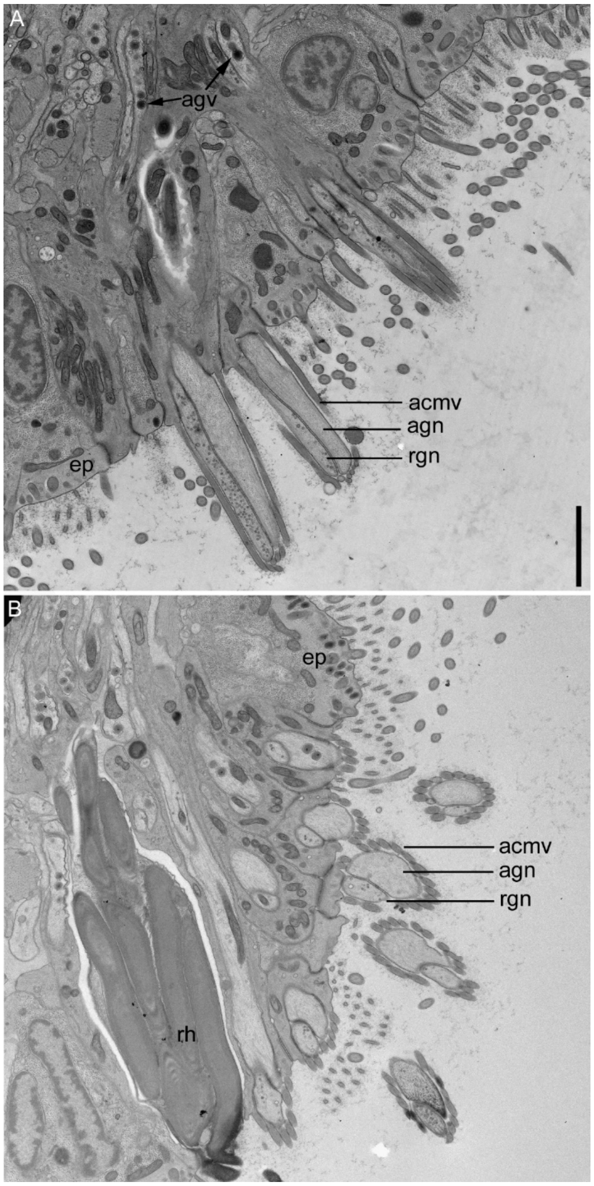

Fig. S14. Empty adhesive glands after mechanical disturbance of animals
(**A, B**) ultrastructural analysis (TEM) of adhesive organs immediately after mechanically disturbing animals for 5 minutes in longitudinal (**A**) and cross section (**B**). Note that no adhesive vesicles can be found in the tips of adhesive glands. However, vesicles are still present in the cell bodies of the adhesive gland cells (**A**, black arrows). Notably, other adhesive organs of the same animals exhibited vesicles in the adhesive gland cell tip. *acmv* anchor cell microvilli, *agn* adhesive gland neck, *ep* epidermis, *rgn* releasing gland neck, *rh* rhabdite. Scale bar: 2 µm.

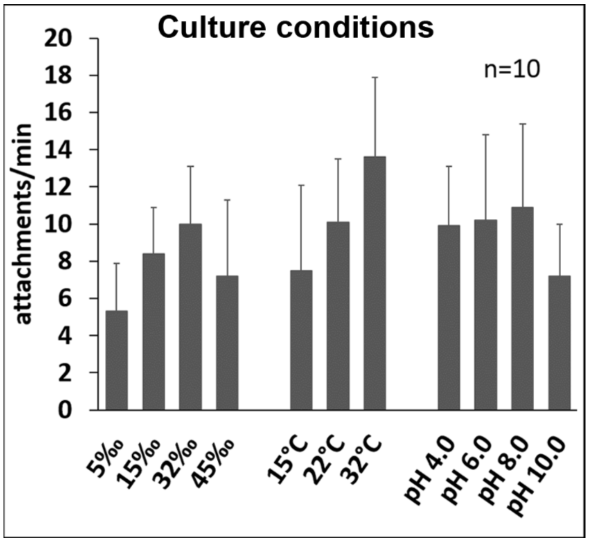

Fig. S15. Attachment ability of *Macrsotomum lignano* in different culture conditions
Average number of attachments per minute under different culture conditions (salinity, temperature, and pH). Scale bars indicate SD (n=10).

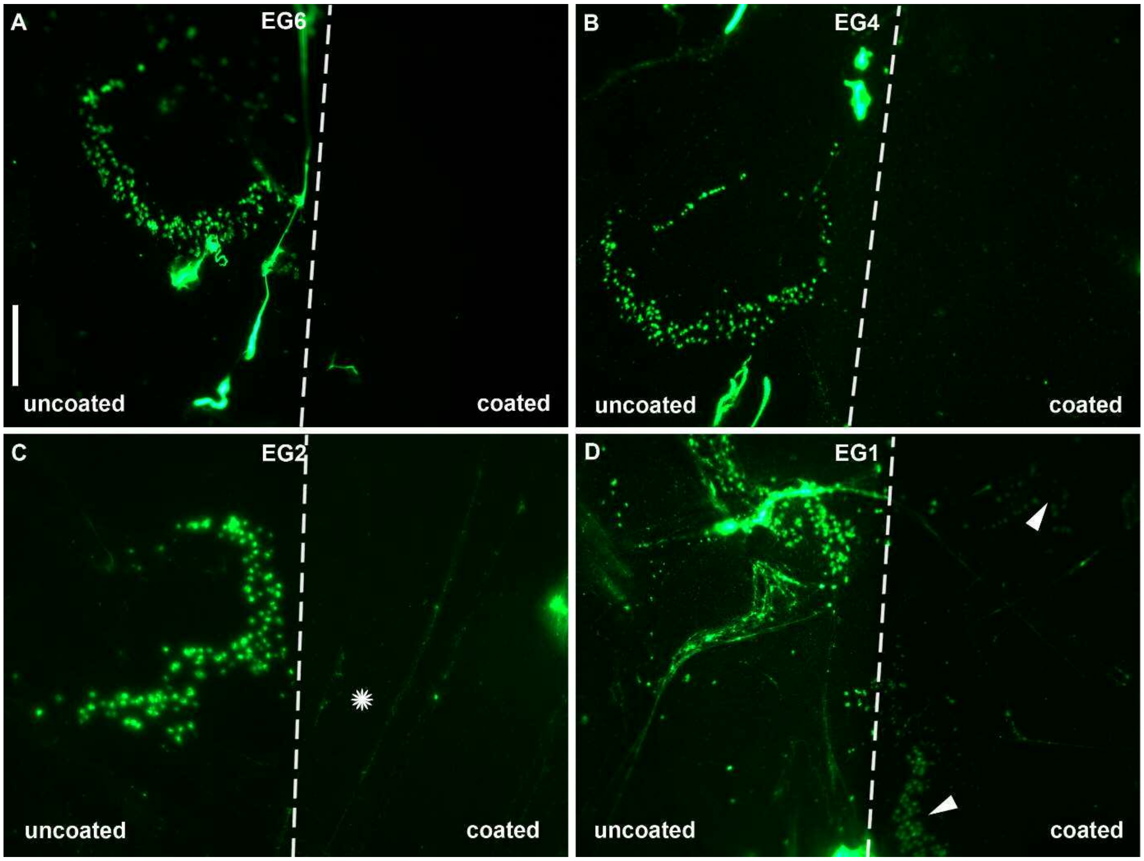

Fig. S16. Adhesion of *M. lignano* with decreasing hydration of surfaces
Fluorescence images of footprints stained with PNA on SAMs with decreasing hydration from EG6 (**A**) to EG1 (**D**). White dotted indicate the border from coated to uncoated area. Note that on EG6, EG4 and EG2 footprints can only be found on the uncoated areas (**A-C**). White stars in **C** indicate adhering mucus on the EG2 coated area. White arrowheads in **D** indicate footprints in EG1-coated area. Scale bar: 50 µm.

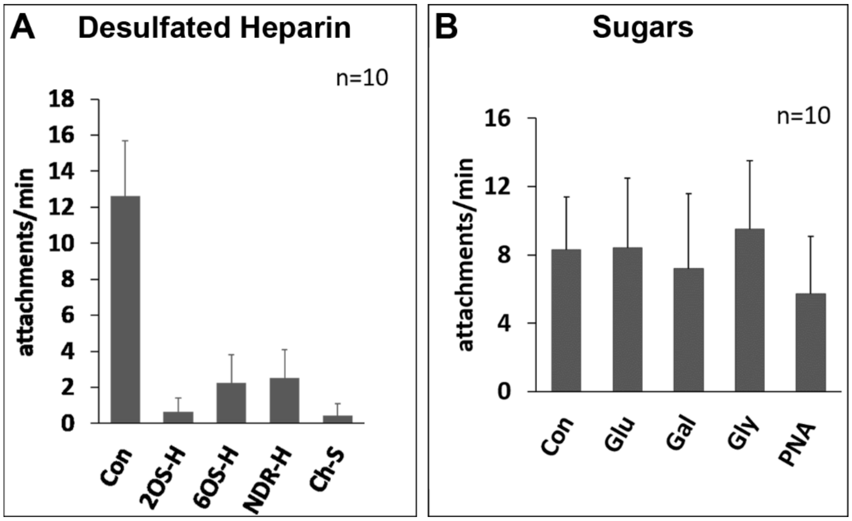

Fig. S17. Interference of attachment with sulfated sugars
(**A**) average number of attachments per minute with 2% of 2-O-desulfated- (2OS-H), 6-O-desulfated- (6OS-H), N-desulfated-re-N-acetylated- (NDR-H) Heparin, and Chondroitin Sulfate (Ch-S), respectively. (**B**) average number of attachments per minute with 10mM Glucose, 10mM Galactose, 100mM Glycine, and 10µg/ml PNA added to the ASW respectively. Scale bars indicate SD (n=10).

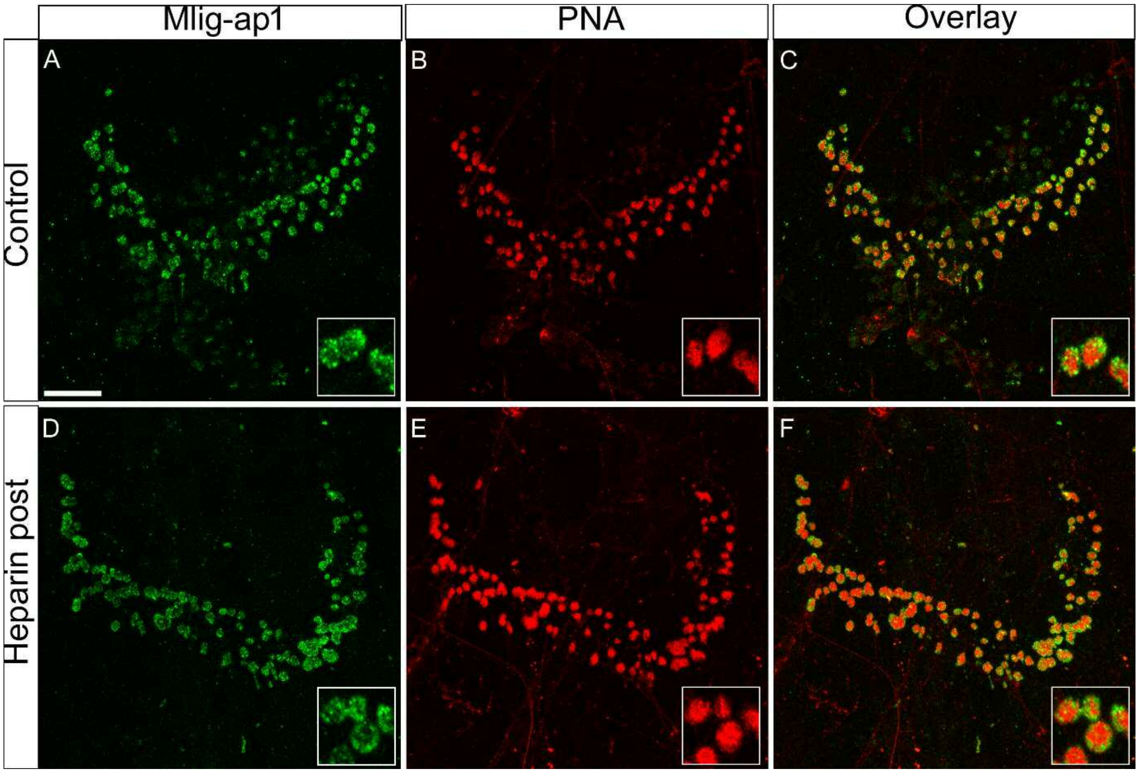

Fig. S18. Heparin treatment on deposited footprints
Confocal projection of double labelling with Mlig-ap1 antibody (green) and for Mlig-ap2 by Lectin PNA (red) on footprints. (**A-C**) control footprints. (**D-F**) footprints were treated with 2% Heparin in ASW. Note that Mlig-ap1 is present - in contrast to footprints where Heparin was added to the medium before footprint collection. Scale bar: 2 µm.

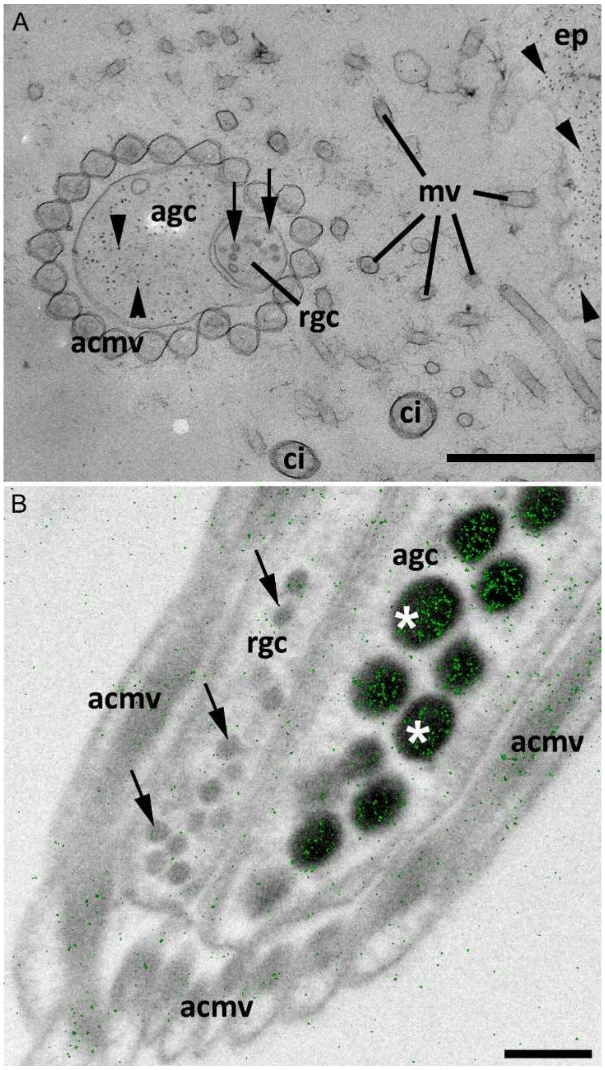

Fig. S19. Labelling of carbohydrates and nitrogen distribution in adhesive gland cells.
Distribution of polysaccharides (**A**) shown by Periodic Acid-Thiocarbohadrazide- Silverproteinate staining (PAS) (TEM). The staining indicates the presence of carbohydrates in the releasing vesicles (arrows). Glycogen (arrowheads) is stained in the epidermal cell and in the adhesive gland cell. (**B**) Recording of nitrogen distribution in false color (green) in ultrathin sections by Electron Spectroscopic Imaging (ESI). Comparatively little nitrogen (i.e. reflecting protein) is present in the releasing vesicles (arrows) in relation to the high nitrogen (i.e. protein) content (green) of the adhesive vesicles (asterisk). *acmv* anchor cell microvilli; *agc* adhesive gland cell; *ci* cilium; *ep* epidermal cell; *mv* microvilli; *rgc* releasing gland cell. Scale bars: (**A**) 1 µm, (**B**) 200 nm.

Table S1. Attachment assays

Attachments per minute upon addition of different chemicals to the culture medium. Conditions and concentrations where animals showed an unnatural overall behavior were not counted (labelled with red).

Category	Individuals	Adhesion within 1 minute: 1	2	3	4	5	6	7	8	9	10	Average	STDEV	Comment
control	control ASW	5	5	10	8	5	8	11	5	6	11	7,4	2,5	
	control ASW	10	5	4	4	3	12	5	4	11	7	6,5	3,3	
	control ASW	13	9	6	6	11	5	9	12	8	4	8,3	3,1	
Sugars	D-Glucose 10-8M in ASW	7	7	10	15	12	8	10	5	3	5	8,2	3,6	
	D-Glucose 10-6M in ASW	4	13	20	9	6	7	6	6	5	3	7,9	5,1	
	D-Glucose 10-4M in ASW	4	13	4	8	9	3	17	18	14	3	9,3	5,9	
	D-Glucose 10-2M in ASW	2	4	12	14	12	11	5	11	5	8	8,4	4,1	
	D-Galactose 10-8M in ASW	7	13	8	9	16	8	10	11	2	3	8,7	4,2	
	D-Galactose 10-6M in ASW	4	8	5	6	2	11	3	9	13	5	6,6	3,6	
	D-Galactose 10-4M in ASW	12	4	4	12	6	3	2	4	3	1	5,1	3,9	
	D-Galactose 10-2M in ASW	17	7	7	8	11	7	5	0	5	5	7,2	4,4	
	D-Galactose 0.1M in ASW	2	6	9	7	7	5	5	5	6	4	5,6	1,9	
	Glycogen 0,05M in ASW													unnatural behaviour
Lectin	PNA lectin 50µg/ml in ASW	5	2	2	4	2	7	4	2	3	8	3,9	2,2	
	PNA lectin 10µg/ml in ASW	7	4	3	3	5	3	1	5	1	2	3,4	1,9	
	PNA lectin 5µg/ml in ASW	11	10	4	6	7	2	9	2	3	3	5,7	3,4	
	PNA lectin 500µg/ml in ASW	2	4	4	2	1	1	2	1	1	1	1,9	1,2	unnatural behaviour
Amino Acids	Glycine 1M in ASW													unnatural behaviour
	Glycine 1M in ddH2O													unnatural behaviour
	Glycine 0.1M in ASW	15	12	6	4	16	8	11	5	9	9	9,5	4,0	
	Glutamate 5% in ASW													unnatural behaviour
	Glutamate 2% in ASW													unnatural behaviour
	Glutamate 1% in ASW	16	18	16	7	11	4	9	14	15	7	11,7	4,8	
Neurotransmitter	GABA 5% in ASW													unnatural behaviour
	GABA 2% in ASW													unnatural behaviour
	GABA 1% in ASW													unnatural behaviour
	GABA 0.5% in ASW													unnatural behaviour
	GABA 0.25% in ASW													unnatural behaviour
	GABA 0.1% in ASW	12	10	4	9	9	12	3	7	14	2	8,2	4,1	
Ab's	Antibody Mlig-ap1 R1 1:10 in ASW													unnatural behaviour
	Antibody Mlig-ap1 R2 1:10 in ASW	12	14	6	8	9	7	12	15	5	11	9,9	3,4	
	Antibody Mlig-ap2 R2 1:10 in ASW	18	7	10	16	5	13	10	6	4	10	9,9	4,7	
Ca	NaCl 33 Promill in ddH2O													unnatural behaviour
	NaCl 33 Promill + CaCl 1.2% in dd H2O													unnatural behaviour
sulfated Charbohydrates	Heparin 0.5% in ASW	3	1	0	0	0	1	1	0	2	0	0,8	1,0	
	Heparin 1.0% in ASW	2	1	0	1	0	1	0	0	0	0	0,5	0,7	
	Heparin 2.0% in ASW	0	0	0	0	1	0	1	0	0	0	0,2	0,4	
	2-O-desulfates Heparin 2% in ASW	0	0	1	2	1	0	2	0	0	0	0,6	0,8	
	6-O-desulfated Heparin 2% in ASW	3	5	2	2	4	1	0	0	3	2	2,2	1,6	
	N-desulfated Heparin 2% in ASW													unnatural behaviour
	N-desulfated-Reacetylated Heparin 2% in ASW	4	3	4	2	0	3	1	5	1	2	2,5	1,6	
	Chondroitin Sulfate 2% in ASW	0	0	1	0	2	0	0	0	0	1	0,4	0,7	

Table S2. Primer list
Primers used for PCR for *in situ* hybridisation Probe synthesis and dsRNA probe synthesis for Mlig-ap1 and Mlig-ap2.

Mlig-ap1		
**Primer Name**	**FW Primer**	**RV Primer**
RNA815_13121.1	ACAAGTGGCTCCACCAACA	CTCGAGCGGTTCGTTTCTCT
RNA815_13121.2	GAGTCACAGTGGCCTCAAGC	ACATGCGACTGTCCTTTTGC
RNA815_13121.3	TTTCCTAGGCTTGTCAATGTGC	TCAGCTAGGCCTTGACATCG
RNA815_317.1	AAGGCCAATTGTATCAACAAACG	AAATTTCTTATTGGCGCACTCG
RNA815_27695.1	AGCGAGGAAGAGTGGACTGAAG	CTATCATGCGCATTTTGGGTAT
RNA815_27695.2	AGTGGATTGACGAAGGAGAAGC	AAGTTGAGACGGGGCAGTAGC
RNA815_13121.1b	TCAGGTAGAAAGTTGACGAAGC	GTCAATGATGGTTGTCTCATCG
RNA815_317.1a	CAGAGGAACGACGGCTACG	AGTCTTTTGGTTTGGTCACAGC
RNA815_317.1c	CTCTGCGAGGACATCAACG	TGTAGCCGTCTCCATAGTAACC

Mlig-ap2		
**Primer Name**	**FW Primer**	**RV Primer**
RNA815_21583	GCGCGTAATAGTTCAAGTTTTGG	ATTCTGGCAGTTGAACTTCTTGC
RNA815_23142	ACCCAGCTGAAACATCAGATTGAG	CTTGTTGTTTAATCGTCGCACTTG
RNA815_300	AAGACTACGGACGATTCACAAGC	CGAAGAACTCTCCGCACTTCC
RNA815_16605a	CTGTGCTGACTACAACCACCAAG	CGATGTCGTAGTACTCTGCGTTG
Mlig013356.g2-A	CAATACCCGAGTCCGAGTACG	TCAATGGGCTTCTTCTGAGG
Mlig013356g2-B	CTAACTTCTTTGCCCTGTGAAGC	CCACCTCCACGAAAGAATCG

Table S3. Advancing and receding contact angles, and ellipsometric thicknesses for the used SAMs.

SAM	Advancing contact angle (deg)	Receding contact angle (deg)	Ellipsometric thickness (Å)
C16	110 ± 1	106 ± 1	20.8 ± 0.5
MHA	32 ± 2	< 10	22.7 ± 0.4
NH2	35 ± 2	< 10	21.2 ± 0.5
EG1	26 ± 1	15 ± 1	24.4 ± 0.4
EG2	31 ± 1	19 ± 1	26.5 ± 0.3
EG4	33 ± 1	25 ± 1	31.4 ± 0.4
EG6	36 ± 1	23 ± 1	37.7 ± 0.5

Movie S1. adhesive papillae are in contact with surface

Attachment behaviour of control *M. lignano*, squeezing preparation of a life animal with detailed view on the tail plate.

Movie S2. RNAi experiment: control animals adhere strongly to substrate
Live observation of RNAi control *M. lignano* after 9 days of tail regeneration

Movie S3. RNAi experiment: Mlig-ap1 RNAi leads to non-adhesive phenotype
Live observation of Mlig-ap1 dsRNA treated *M. lignano* after 9 days of tail regeneration

Movie S4. RNAi experiment: Mlig-ap2 RNAi leads to non-adhesive phenotype
Live observation of Mlig-ap2 dsRNA treated *M. lignano* after 9 days of tail regeneration

Movie S5. RNAi experiment: double RNAi leads to non-adhesive phenotype
Live observation of Mlig-ap1 and Mlig-ap2 dsRNA treated *M. lignano* after 9 days of tail regeneration

Movie S6. Adhesive organs are used sequentially
Live observation of the attachment in a squeezed animal with detailed view on the tail plate

Movie S7. MgCl2 hinders the release
Live observation of *M. lignano* in 80% $MgCl_2$ in ASW

Movie S8. 1-Phenoxy-2-Propanol does not impair the release
Live observation of *M. lignano* in 0,1% 1-Phenoxy-2-Propanol in ASW

Movie S9. Arginine and Lysine hinder the release
Live observation of *M. lignano* in 8% Arginine and 3% Lysine in ASW

Datasets D1, D2, D3 (separate files)

Mass spectrometry analyses
Datasets D1 - D3 are Excel files (Mass Spectrometry analyses output) showing peptides found in different mass spectrometry analyses. Single experiments are displayed on different sheets. Mlig-ap1 and Mlig-ap2 are colored in yellow in all files.

References

1. Arbore R, et al. (2015) Positional RNA-Seq identifies candidate genes for phenotypic engineering of sexual traits. *Front Zool* **12**:14.

2. Lengerer B, et al. (2018) Organ specific gene expression in the regenerating tail of Macrostomum lignano. *Dev Biol* **433**:448–460.

3. Weber M, et al. (2018) A targeted in situ hybridization screen identifies putative seminal fluid proteins in a simultaneously hermaphroditic flatworm. *Bmc Evol Biol* **18**:81.

4. Lengerer B, Hennebert E, Flammang P, Salvenmoser W, Ladurner P (2016) Adhesive organ regeneration in Macrostomum lignano. *Bmc Dev Biol* **16**:20.
